# The Epidemiology and Demographics of Hip Dysplasia

**DOI:** 10.5402/2011/238607

**Published:** 2011-10-10

**Authors:** Randall T. Loder, Elaine N. Skopelja

**Affiliations:** ^1^Section of Orthopedic Surgery, Riley Hospital for Children, ROC 4250, 705 Riley Hospital Drive, Indianapolis, IN 46202, USA; ^2^Department of Orthopaedic Surgery, Indiana University, Indianapolis, IN 46202, USA; ^3^Ruth Lilly Medical Library, Indiana University School of Medicine, Indianapolis, IN 46202, USA

## Abstract

The etiology of developmental dysplasia of the hip (DDH) is unknown. There are many insights, however, from epidemiologic/demographic information. A systematic medical literature review regarding DDH was performed. There is a predominance of left-sided (64.0%) and unilateral disease (63.4%). The incidence per 1000 live births ranges from 0.06 in Africans in Africa to 76.1 in Native Americans. There is significant variability in incidence within each racial group by geographic location. The incidence of clinical neonatal hip instability at birth ranges from 0.4 in Africans to 61.7 in Polish Caucasians. Predictors of DDH are breech presentation, positive family history, and gender (female). Children born premature, with low birth weights, or to multifetal pregnancies are somewhat protected from DDH. Certain HLA A, B, and D types demonstrate an increase in DDH. Chromosome 17q21 is strongly associated with DDH. Ligamentous laxity and abnormalities in collagen metabolism, estrogen metabolism, and pregnancy-associated pelvic instability are well-described associations with DDH. Many studies demonstrate an increase of DDH in the winter, both in the northern and southern hemispheres. Swaddling is strongly associated with DDH. Amniocentesis, premature labor, and massive radiation exposure may increase the risk of DDH. Associated conditions are congenital muscular torticollis and congenital foot deformities. The opposite hip is frequently abnormal when using rigorous radiographic assessments. The role of acetabular dysplasia and adult hip osteoarthritis is complex. Archeological studies demonstrate that the epidemiology of DDH may be changing.

## 1. Introduction

Demography is the study of human populations with reference to size, diversity, growth, age, and other characterizing statistics [1]. Epidemiology is the study of the incidence, distribution, and determinants of disease frequency in groups of individuals who happen to have characteristics in common (e.g., gender, ethnicity, exposure, genetics) [2, 3]. Incidence is the proportion of new cases in the population at risk during a specified time interval; prevalence is defined as the proportion of individuals with the disease in the study population of interest. Demographic and epidemiologic studies can determine risk factors for a disease/condition of interest, shed light on its etiology, and guide potential prevention programs. 

Developmental dysplasia of the hip (DDH) is an epidemiologic conundrum [4]. DDH encompasses a wide spectrum of pathology ranging from a complete fixed dislocation at birth to asymptomatic acetabular dysplasia in the adult [5–9]. The epidemiologic literature regarding DDH is vast and confusing due to different definitions of hip dysplasia, different methods of diagnosis (e.g., physical exam, plain radiographs, ultrasound), different ages of the population studied (e.g., new born, 1 month old, 3 months old, etc.), clinical experience of the examiner [10], different ethnicities/races in the examined population, and different geographic locations within similar ethnic populations [11, 12]. Neonatal hip instability, now even more apparent with hip ultrasonography, must also be addressed [13, 14]; the clinical challenge is to separate the neonatal hip instability which resolves spontaneously from that which is significant [15–21]. 

The last major review of the epidemiology of hip diseases was in 1977 [22]. The goal of these manuscripts is to update the current knowledge of the epidemiology and demographics of pediatric hip disease which may lead to significant morbidity in later life. 

## 2. Materials and Methods

A systematic review was performed for articles on DDH in infants focusing on etiology, epidemiology, and diagnosis. Exclusion criteria were those manuscripts discussing surgery, therapy, rehabilitation and any foreign language articles without an English abstract. There were certain difficulties in searching the literature on this topic because of the many variant names for DDH. The most commonly used modern terms are “developmental dysplasia of the hip” or DDH and “congenital hip dislocation,” CDH. Archaic terms include “congenital dislocation,” “congenital hip,” or “congenital subluxation of the hip” or “congenital dysplasia of the hip.” Even with controlled vocabularies, each database uses a different subject term, for example, Medline's (Medical Subject Headings or MESH) heading is “Hip Dislocation, Congenital,” EMBASE uses “Congenital Hip Dislocation,” Web of Science uses “Congenital Dislocation,” and the historical Index-Catalogue uses “Hip Joint, Dislocation of, Congenital.”

The databases used in this paper were PubMed Medline (1947–2010) (http://www.ncbi.nlm.nih.gov/pubmed/), Ovid Medline (1947–2010), EMBASE (1987–2010), WorldCat (1880–2010) (books and theses) (http://firstsearch.oclc.org/), Web of Knowledge (1987–2010), and IndexCat (Index Catalogue of the Library of the Surgeon-General's Office (1880–1961) (http://www.indexcat.nlm.nih.gov/). Individual orthopedic journals were also searched for articles published prior to 1966 that predate electronic indexing, including *Journal of Bone and Joint Surgery (American and British), Clinical Orthopaedics and Related Research*, and *Acta Orthopaedica Scandinavica*. Hand searching and citation searching were also performed. Google Scholar (1880–2010) (http://scholar.google.com) was searched as a final check but did not find any additional articles. Age groups were limited to those <18 years old; duplicate citations were removed.

This search resulted in 2277 unique manuscripts which were reviewed to find those that discussed any of the topics regarding DDH and epidemiology, etiology, demographics, incidence, prevalence, race, gender, family history, inheritance, genetics, age, bone age, weight (either birth weight or normal weight), height, growth, maturation, any other anthropometric characteristics, seasonal variation, hormone, endocrine, congenital anomalies, perinatal factors, swaddling, collagen, and opposite hip. Of these 2277 manuscripts, 422 provided ample information and are the contents of this paper.

## 3. Results

### 3.1. Childhood Hip Dysplasia

**Incidence**

**Figure 1:**
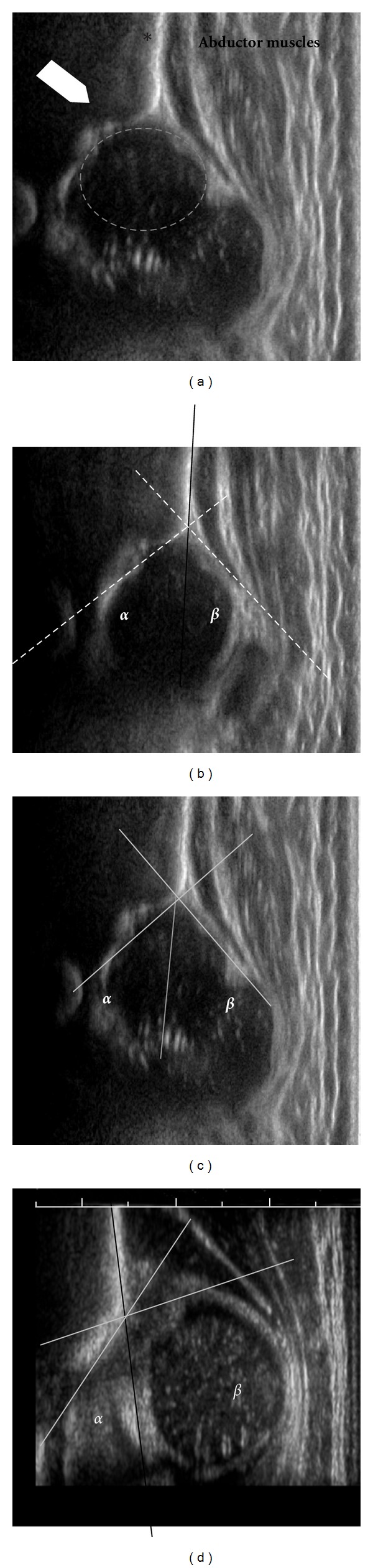
Ultrasound neonatal hip examination. (a) A representative longitudinal ultrasound image of a normal neonatal hip. The ilium is marked by the asterisk, the bony acetabular roof by the large arrowhead, and the abductor muscles seen by the longitudinal white/gray alternating structures. (b) Measurement of the alpha (*α*) and beta (*β*) angles on ultrasound establish the Graf class. The baseline is first drawn and is the line along the ilium as it intersects the bony and cartilaginous portions of the acetabulum (solid black line). The *α* angle is the angle between the baseline and the roof of the bony acetabulum; the *β* angle is the angle between the baseline and the cartilaginous roof. (c) An example of a Graf IIc hip, with an *α* angle of 43° and a *β* angle of 49°. (d) An example of a Graf IV hip, irreducible dislocated hip, with an *α* angle of 42°. Typically *β* angles are not measured on dislocated hips, but in this example it would measure 99°.

There are three eras in modern medicine when the incidence of hip DDH has been determined [26]. Period I (1920s to 1950s) was when the incidence was arbitrarily estimated. Period II (1950s to 1980s) was when the incidence was determined based on the detection of unstable hips on neonatal physical exam plus the addition of late-diagnosed patients. Period III (1980s to present) incorporates hip ultrasonography (both diagnostic and screening) (Figure 1). Generally accepted ranges for the incidence of DDH for Period I range from 0–40% (0% Africans and up to 40% in other racial groups), for Period II 0.04%–16.8% (0% in Africans and up to 33% in Native Americans), and for Period III 4.4%–51.8% (4.4% for Africans and 7.15% the lowest for Caucasians) [26]. The wide range of DDH incidence in Period III reflects differences in the definition of DDH. Some studies include any hips with ultrasonographic instability, whereas others include only those types ≥ Graf IIb (Table 1, Figure 1).

Hip ultrasonography creates confusion due to differences between the neonatal physical exam and ultrasound findings [15–17, 27, 28]. The interobserver variability (*κ* 0.4 to 0.5) in determining hip stability is also poor to moderate [29–31]. The wide variability in incidence in the ultrasonographic era is better understood when considering two different groups of neonatal instability. The first is neonatal, sonographic DDH which resolves spontaneously. The second is neonatal instability which if left untreated may progress to true DDH, ranging from acetabular dysplasia to complete dislocation. Hadlow [32] noted that 50% of unstable hips at birth stabilized completely within 5 days; Barlow [33] noted that 90% of unstable hips at birth become normal by 2 months; Abdinejad et al. [34] noted that 97% of neonatally unstable hips resolved spontaneously by 6 months. Many of the sonographic DDH hips likely reflect these clinical findings [20, 32–34] and are in the first group.

The incidence/prevalence is quoted as the number per 1000 live births. The data presented here is a best attempt at synthesizing the literature during these different periods; insufficient data was often present making it difficult to calculate demographic variables. Race is classified using the definitions of Eveleth and Tanner: Caucasians, Africans in Africa and of African Ancestry, Asiatics (Amerindians, Hispanics, Indonesian-Malays), Indo-Mediterraneans (inhabitants of the Near East, North Africa, and Indian subcontinent), and Australian Aborigines and Pacific Island peoples [35].

### 3.2. Clinical Screening Period (1950s–1980s) by Ethnic Groups (Table 2) 

#### 3.2.1. Conventional DDH

**Indigenous Peoples (Table 2(a))**

**(i) Native Americans**

The incidence of DDH is high in Native Americans, likely due to a combination of genetics and swaddling. In the Arizona Fort Apache Indians [43], the incidence was 31, but likely higher, since cases of dysplasia and subluxation were excluded. This particular group of Native Americans represent a very tight gene pool as they have maintained their endogamous marriages (band members only marrying within their own band) after migrating approximately 600 years ago from three different bands in Western Canada. In Navajo children [42] from Fort Defiance, Arizona and Gallup, New Mexico, the incidence was 67. A positive family history was present in 33% of the dysplasia cases but only 6.1% of the nondysplastic cases. In the Navajo from the Arizona Many Farms District, [41] the overall prevalence of DDH was 37.9 : 33.3 for adults and 40.1 for children. Complete dislocation was more common in adults and simple acetabular dysplasia/subluxation more common in children. The ratio of childhood dysplasia to dislocation was 4.5 to 1 and in adults 0.3 to 1.

**Figure 2:**
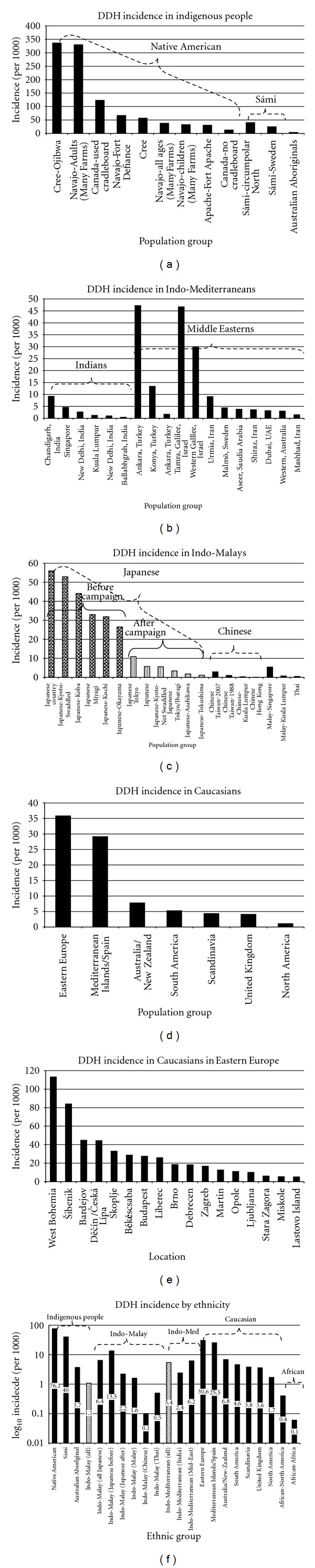
The incidence of DDH in various ethnic groups. (a) The incidence of DDH in indigenous populations. (b) DDH incidence in Indo-Mediterraneans. (c) DDH incidence in Indo-Malay peoples. (d) DDH incidence in all Caucasians. (e) DDH incidence in Eastern European Caucasians. (f) Incidence of DDH amongst all ethnic groups; note the *y*-axis is logarithmic_10_.

In an early study of the Cree-Ojibwa, Island Lake, Northern Manitoba, the incidence was 36 [36]; in a later more detailed study, the incidence of frank dislocation and subluxation was 110 [37]. The prevalence of DDH for all ages was 336 [37] (195 for frank hip dislocation or subluxation, 99 for dysplasia, and 54 for other types). In the Cree in Northern Saskatchewan, the overall prevalence was 13.2 [38]. In Ontario Native Americans [39], the incidence ranged from 12 to 123. Using weighted averages, the average incidence of DDH in Native Americans is 76.1 for all dysplasia (Figure 2(a)).

**(ii) Sámi and Australian Aboriginals**

The Sámi (previously known as “Lapps” which is a derogatory offensive term) is the indigenous people of Sápmi, the circumpolar areas of Sweden, Norway, Finland, and the Kola Peninsula of Russia [151]. The Sámi population is 50,000 to 100,000, and ~1/2 live in Norway [151–153]. The incidence of DDH in the Sámi was 24.6 [46] and 40 [45]. The incidence in Australian Aborigines is ~1/2 that of Caucasians (3.7 versus 6.6) [44] (Figure 2(a)).

**(iii) Africans**

DDH is extremely rare in Africans (Table 2(b)). In Sub-Saharan Africans, 2 cases of typical DDH were described in the Bantu [48]. There were no signs of hip dysplasia at 3 months of age in another study of 16678 Bantu children [47], despite breech presentation in 897 (5.4%). In the Kikuyu Bantu, Kenya, 2 cases of typical DDH are described [154]. In a review of 284 children with congenital orthopaedic malformations in an African teaching hospital (Ibadan, Nigeria), DDH accounted for only 2.2% of all congenital malformations [155].

This immunity of the African infant from DDH may be due to deeper acetabulae [156], genetic factors [157], and the absence of swaddling in African cultures. Carrying the infant in an abducted position straddling the iliac crest is postulated as protective against DDH in the African peoples. However, in the United States, the acetabular indices of Caucasian and African infants showed minimal differences at birth but by 6 to 12 months of age were actually slightly higher (or shallower acetabulae) in Africans [158]. Genetic mixing between Africans and other races with a higher incidence of DDH (e.g., Caucasians in the United States) [48, 159] results in a higher but still comparatively low incidence of DDH. Quoted incidences in African Americans are 0.21 [51] and 0.46 [50] compared to 1.5 in American Caucasian infants [50]. Using weighted averages, the incidence of DDH is 0.06 in Africans in Africa and 0.40 in the United States.

**(iv) Indo-Mediterraneans**

The incidence in India is 0.42 in rural Ballabgarh, Haryana [52], 1.0 [53] and 2.65 [55] in New Delhi, and 9.2 in northern India (Chandigarh) [57]. For Indians in Malaysia, it is 1.2 [54] and 4.63 in Singapore [56]. In Iranians, it is 1.5 in Mashhad City [65], 3.64 in Shiraz [34], and 9.1 in Urmia [66]. In Dubai, UAE, the incidence is 3.17 [62] and 3.8 in Aseer, Saudi Arabia [63]. In Western Galilean Arabic's it ranges from 30.0 to 46.7 [61]. In Ankara, Turkey, it is 1.7 [58] and 47 [60], and 3.42 in Konya, Turkey. Using weighted averages, the incidence of DDH in Indo-Mediterraneans is 5.4, 2.4 for those of Indian descent, and 6.2 for those of Arabic descent (Table 2(b), Figure 2(b)).

**(v) Indo-Malays**

The incidence of DDH in Indo-Malays varies widely (Table 2(c), Figure 2(c)). In Japanese the incidence ranges from 1.8 [87] to 52.9 [85]; for Chinese 0.07 [74] to 4.41 [56]; for Malay 0.71 [54] to 5.38 [56]. The one study of Thai note an incidence of 0.51 [75]. Using weighted averages, the overall incidence of DDH in Indo-Malays is 1.1, 0.1 in Chinese, 1.6 in Malay, and 6.4 in Japanese.

**(vi) Caucasians**

**(a) Europe**

The incidence in Scandinavia ranges from 0.9 to 28 [51, 64, 92, 94–102, 104–106, 160]. In the United Kingdom, three studies give a low incidence (0.91 in Birmingham, England [111], 1.55 in Manchester, England [33], and 1.7 in Northern Ireland [118]); most range from 3–6 [107, 109, 112–115, 161, 162], with the highest incidence of 30.3 in Aberdeen, Scotland [108]. In Spain, the incidence was 9.78 in Córdoba [121] and 43.4 in Madrid [122] (5.09 for complete dislocation). In the Mediterranean Islands, it was 10.6 in Crete [124] and 25.5 in Sicily [125]). The incidence of DDH is higher in Eastern Europe and ranges from 5.2 in Lastovo Island, Croatia [128] to 113 [133] in West Bohemia, Czech Republic. The average weighted incidence of DDH in the Scandinavia is 3.8, 3.6 in the United Kingdom, 25.5 in Spain and the Mediterranean Islands, and 35.8 in Eastern Europe (Table 2(d), Figure 2(e)).

**(b) Australia/New Zealand**

The incidence is 7.7 in Adelaide [144], 5.5 in South Australia [67], and 6.6 [44] and 10.5 [143] in Western Australia. In New Zealand, it is 2.19 in Southland [147], 2.65 in Canterbury [146], 3.54 in Auckland [145], and 16.0 [32] in New Plymouth. The averaged weighted incidence of Caucasians in Australia/New Zealand is 6.8.

**(c) Americas**

There are few incidence studies in the United States due to its highly mobile population. The incidence is 0.7 in Jefferson County, Alabama [51], 0.8 in Portland, Oregon [150], 1.1 in Iowa [163], and 0.7–6.1 in Utah [164]. In Llanquihue, Chile [149], the incidence is 4.6, 2.3 for complete dislocation and 2.2 for dysplasia/subluxation. The incidences for all Caucasians are shown in Figures 2(d) and 2(e).

**(vii) Mixed Races**

In a study of 432778 infants born in Birmingham, England between 1960–1984 [165], the birth prevalence of DDH was 2.77 when both parents were Caucasian, 1.37 when both were South Asian (from India, Pakistan, Bangladesh), and 0.66 when both were Caribbean (primarily African). These numbers are similar to the average weighted incidences in this study (3.6 for the United Kingdom, 2.4 for Indian, and 0.1 for Africans), the value for Caucasians and South Indians slightly lower than ours, while that for the Africans is slightly higher. These values changed with mixed matings; 2.77 to 0.78 when one parent was Caucasian and one South Indian and 0.66 to 1.28 when one parent was Caucasian and one Caribbean/African. This confirms the differences noted in the United States with genetic mixing in Africans. Other incidence figures for mixed or unknown racial groups are shown in Table 2(b).

#### 3.2.2. Clinical Neonatal Hip Instability (Table 3)

**Indigenous Peoples**

The incidence of neonatal hip instability in the Maori is less than Caucasians [191], where 16% of the births in one hospital were Maori, but only 7% of the DDH cases were Maori.

**(i) Africans**

In Africans, the incidence was 0 in North African Ethiopian Jews [204], 0.3 in South Africa [49], and 2.0 in Uganda [166]. In Oklahoma City it is 0.4 [167] and 0.42 in New York City [168].

**(ii) Indo-Mediterraneans**

The incidence of neonatal hip instability is 0.17 in Mumbai [205] and 18.7 in New Delhi, India [55], 1.25 in Kuwait (primarily Palestinian) [169], 4.9 in Dammam, Saudi Arabia [170], and 36.5 in Abha, Saudi Arabia [171].

**(iii) Amerindians**

The incidence of neonatal hip instability in Guanajuato, México is 1.47 [172].

**(iv) Indo-Malay**

The incidence in Taiwan is 1.2 in Taichung [175] and 1.8 in Taipei [174].

**(v) Caucasians**

In Europe, the incidence of neonatal hip instability is 4.1 in Uxbridge, England [161], 5.65 in Falköping, Sweden [178], 7.7 in Dublin, Ireland [185], 10.2 in Västerbotten County, Sweden [96], 10.2 in Uppsala, Sweden [180], 12.8 in Cork, Ireland [206], 19 in Bristol, England [187], 20.4 in Uppsala, Sweden [181], 32.2 in Leipzig, Germany [189], 50.0 in Aberdeen, Scotland [188], and 61.7 in Poland [190]. In Australia/New Zealand, it is 3.4 and 8.5 in Auckland, New Zealand [191], 6.6 in Western Australia [192], 6.7 in Sydney, Australia [193], and 19.4 in Victoria, Australia [194]. In North America, it is 1.4 in Iowa City [196], 5.2 in Oklahoma City [167], 8.6 in Salt Lake City [195], 9.2 in Indianapolis, Indiana [198], 5.7 [207] and 9.9 in Vancouver, British Columbia [197], and 15.3 in New York City [168]. The average weighted incidence for all Caucasians is 10.8 (6.0 in Australia/New Zealand to 23.2 in Western Europe).

#### 3.2.3. Ultrasonographic and Clinical Screening Period (1980s to Present) (Table 4)

For this paper, sonographic DDH is defined as a hip > Graf IIa. North African Black infants (Ethiopian Jews) have a sonographic incidence of 12.4 at birth and 1.5 at 4 to 6 weeks [157]. In Africans living in London, the incidence of sonographic DDH was 0 (0 of 185) [208]. Caucasian infants demonstrate a sonographic incidence at birth from 7.6 [64] to 847 [216], with a weighted average of 131. At 4 to 6 weeks of age, this drops to 12.8–14.8, with a weighted average of 14.1. In Turkey, the incidence of sonographic DDH at 6 weeks is 47.1 [60]. 

Composite results (Table 4) denote an average incidence of ultrasonographic DDH in Caucasians at birth of 80.0 (7.6 to 847) and 42.2 (3.8 to 103) for hips > Graf IIa (range). At 4 to 6 weeks of age, these numbers drop to 52.9 (range 3.1–379.4) and to 29.5 for hips > Graf IIa, and by 4 to 6 months of age to 7.5–34.7 for DDH > Graf IIa. This incidence of 7.5–34.7 is similar to that for Caucasians during the clinical screening period from 1950–1980 (3.8 in Scandinavia, 5.5 in Western Europe, 6.8 in Australia/New Zealand, and 35.8 in Eastern Europe) (Table 2).

### 3.3. Gender, Laterality, Family History, Perinatal Factors (Table 5)

**Figure 3:**
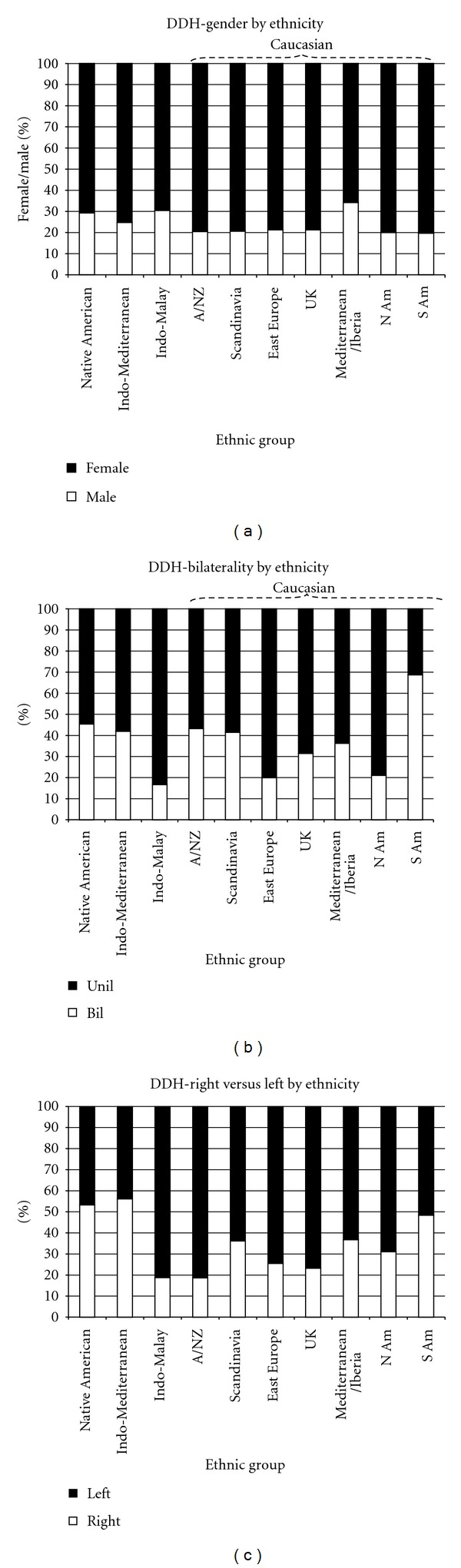
Variability in DDH demographics amongst ethnic groups. (a) Variability in gender amongst ethnic groups. (b) Variability in unilateral/bilateral involvement amongst ethnic groups. (c) Variability in right and left hip involvement amongst ethnic groups.

Typical risk factors for DDH are said to be female, first born, breech position, positive family history, left hip, and unilateral involvement. In 9717 cases (Table 5), 75.5% were female and 63.4% unilateral. When unilateral, 36.0% involved the right and 64.0% the left hip. Left-sided predominance of DDH may reflect the finding that right-sided laterality in birth defects correlates with the proportion of males among a group of infants with any given pathology [232]. There is minimal gender variability by ethnicity (Figure 3(a)) but considerable variability in bilaterality (Figure 3(b)), ranging from 16.7% in Indo-Malay to 69% in South American Caucasians. Although the left hip is typically more involved in those with unilateral dysplasia, there is significant ethnic variability, 44% in Indo-Mediterraneans to 81.4% in Caucasians from Australia/New Zealand (Figure 3(c)). The prevalence of mild adult acetabular dysplasia in children with documented unilateral DDH is up to 40% [233]. 

Breech position/presentation increases the incidence of DDH [32, 34, 44, 54, 56, 63, 65, 67, 100–102, 105, 106, 114, 118, 135, 139, 162, 168–171, 191, 225, 226, 229–231, 234–243]. Breech position/presentation in children with DDH ranges from 7.1% [32] to 40% [65]. In Western Australia [44], the incidence was 27.7 for breech and 5.5 for vertex presentations; in Denmark [106], 18.9 for breech and 5.5 for vertex presentation; in Northern Ireland, 6.94 for breech and 1.55 for vertex presentation [118]. In Singapore, the incidence was 10.7 in breech deliveries, 8.4 in vacuum extraction deliveries, and 0.7 overall [54]. In Norway [100–102], 15.7% of DDH children were breech compared to 3.4% in the normal population; in Helsinki, Finland, these numbers were 19.0% and 3.5% [105]; in Hungary, 11.4% and 3.1% [142]. In Riyadh, Saudia Arabia, these same numbers were 38% and 8.8% [226]; in Kuwait, 7% and 3.7%. In two Finnish hospitals, DDH was present in 2.6 and 6.6% of children with breech presentation [240], 7.7% in Stockport, England [229], 18% in Scotland [162], and 25% in London [235]. In Southampton, England, 36% of complete dislocations and 83% of subluxations were breech [112]. In certain Native Americans, there is no correlation with breech presentation/delivery [37, 44]. 

Breech presentation/presentation also influences neonatal hip instability. In 6571 live births (257 breech) [244], the odds ratio (OR) of hip instability was 3.42 in all breech babies and 11.1 for those with DDH needing treatment. The incidence of clinical hip instability in breech babies is 44 in Norway [179] (61 specifically in Trondheim, Norway [245]), 71 in New York City [168], 107 in Thailand [236], 131 in Leipzig, Germany [189], and 260 in Malmö, Sweden [176]. In Leipzig [189], the incidence of neonatal hip instability was 131 in breech and 30 in vertex presentations; in Tiachung, Taiwan, these numbers were 8.9 breech and 0.6 vertex [175]. In Dammam, Saudi Arabia [170], breech presentation was present in 13% of newborns with neonatal hip instability and 2.1% without. In Trondheim, Norway, the incidence of ultrasonographic hip instability in breech presentation is 61 [245]. In Germany, the incidence of ultrasonographic (> Graf IIa) neonatal hip instability in 3739 newborns was 136 in 317 breech children and 64 in nonbreech children [242]. In another German study; however, there was no correlation between intrauterine presentation and sonographic hip instability [215]. 

Breech-type (frank breech or bilateral hip flexion/knee extension, nonfrank, or varying amounts of hip and knee flexion) is also important. The incidence of DDH in Hungarian breech children was 340 in nonfrank and 185 in frank breech [241]. In Norway [245], the incidence of DDH in frank breech was higher than other breech types. DDH in breech children may be decreased by elective Caesarean section [246]; of 941 breech presenting infants, the incidence of DDH was 3.69% (19 of 515) when delivered by elective pre-labor Caesarean section, 6.64% (26 of 241) when delivered by intrapartum Caesarean section, and 8.11% (15 of 185) when delivered vaginally. In New York, children born by Caesarean section had a 3.4 times higher chance of DDH when breech compared to vertex presentation, and those born vaginally had a 7.0 times higher chance of DDH when breech compared to vertex presentation [168]. 

The incidence of DDH is less in premature and low-birth-weight infants [44]. Children <37 wk gestational age had an incidence of 3.4, 37–42 wks 6.3, and >42 wks 29.9; for those <2500 gms, the incidence was 4.1, 2500–3499 gms 6.7, and >3500 gms 6.4. In another study, all babies with DDH had a gestational age of 39 weeks or more, and 52% were firstborn [234]. In Northern Ireland, the risk of DDH was less when the birth weight was <3.0 kg [118]. Children born large for gestational age (LGA) have an increased risk of hip subluxation (OR 1.73) [247] and twice the incidence of ultrasonographic DDH (> Graf IIc) compared to normal-birth-weight newborns (6.1% versus 3.5%) [248]. In breech presentation, DDH is more common in those with higher birth weights (3.49 kg with DDH, 3.06 kg without DDH) [245]. Very-low-birth-weight infants are not at increased risk of DDH [249]. 

Primiparity increases the risk of DDH. In Hungary [142], the average birth order of 1767 children with DDH was 1.37 compared to 1.54 for 108966 control children showing that DDH children are skewed to the first born. In Finland, 63% of DDH children were first born compared to 55% in the normal population [105], in Southampton, England, 83% of children with subluxation were first born [112], and in Madrid, Spain, 50% of were firstborn [122]. In Western Australia [44], the incidence was 7.6 if first born and 5.9 if multiparous. At Christchurch Women's Hospital, first-borns accounted for 83% of DDH children but only 42% of all births [146]. In Utah, USA, the primiparity was 46% greater than expected in the 327 cases of DDH. In Singapore [56], 43.8% to 50% of DDH children were firstborn [54].

A positive family history increases the risk of DDH [38, 42, 60, 63, 65, 105, 162, 225, 230, 250–253]; it was 14% in Poland [253], 21% in Saudia Arabia [63], and 35% in Greece [124]. In Native Americans, it was 33% in the Navajo [42]. In northern Saskatchewan Cree, it was 16% in sisters and 14% in mothers [38] but no correlation in the Manitoba Cree-Ojibwa [37]. In 589 English children with DDH, 4.01% of 1st degree and 0.33% of 2nd and 3rd degree relatives had DDH [162]. In 1256 Japanese children with DDH, 6.1% of siblings, 0.7% of parents, and 0.5% of uncles/aunts had DDH [252]. In 500 Utah children with DDH, 24.5% of 1st degree relatives had DDH [164]. In two Hungarian families with DDH, DDH occurred in 14% of family members: siblings, 2.1–2.3%, parents, 1.2–1.4% of uncles/aunts, and 4.7% of cousins [254]; recurrence risks were 8, and 4x increased in brothers and sisters, 4x in parents, 2.5x in uncles/aunts, and 2.0–2.5x in cousins compared to the general population. The risk or liability of inheriting DDH amongst siblings was 49% in Turkey [255]; the overall heritability was 74% in Norway [101]. An association between DDH and familial primary acetabular dysplasia [256] also exists; radiographs of the mothers of DDH children who did not have any known preexisting DDH demonstrated acetabular dysplasia in 8.65% [252]. One negative study exists regarding the association between ultrasonographic DDH and family history [215].

Consanguinity results in a very high incidence of DDH in Japan [81] and the Middle East where 25% [169], 40% [65], and 49% [225] of DDH cases were from consanguineous parents. In western Galilee, an area with a high incidence of DDH, marriage between 1st cousins is frequent [61, 257]. Tight gene pools were implicated in the high incidence of DDH in Fort Apache Navajos [43]. 

To simplify these figures, epidemiologists use decision analysis/meta-analysis or multiple logistic regression to determine DDH risk factors. The clinical practice guidelines of the American Academy of Pediatrics [5] used a decision analysis model, concluding that the baseline incidence of DDH (not at risk children) was 11.5 (4.1 for boys and 19 for girls). The relative risk with a positive family history is 1.7 times higher (for an absolute incidence of 6.4 for boys and 32 for girls), and the relative risk for a breech presentation compared to vertex is 6.3 times higher (for an incidence of 29 for boys and 133 for girls). 

Logistic regression analysis of 1127 South Australian DDH children (1986–1993) [7] demonstrated that breech presentation, oligohydramnios, female gender, and primiparity were significant positive risk factors for DDH; low birth weight and prematurity were protective. The ORs were 17.2 for breech deliveries, 10.0 for breech presentation with Caesarean delivery, 4.0 for oligohydramnios, 3.9 for female gender, 2.7 for very high birth weight (>4500 gm), 2.2 for first born, and older maternal age (1.71 for 30–34 years old and 1.72 for ≥35+ years old). Protective ORs were low birth weight (0.3 for <2000 gm, 0.52 for 2000–2499 gms) and prematurity (0.42 for <37 wks gestation, 0.59 for 37 wks gestation, and 0.80 for 38 wks gestation). 

In 51 Israeli DDH children (1994-1995), ORs were 6.0 for breech presentation, 4.3 for female gender, and 2.7 for primiparity [258]. In 81 Nottingham children with DDH and 323 control subjects [259], the ORs for those born by Caesarean section or breech delivery were 3.29 and 4.57, respectively, and 0.55 and 0.53 for multigravida and multiparity; maternal age, gestational age, placental weight, race/ethnicity, and birth weight/height were not risk factors [259].

In 1,059,479 Norwegian children [179], predictors of neonatal hip instability (clinical exam) were gestational age, gender, and breech presentation; birth order had no effect. The overall incidence of neonatal hip instability overall was 10.3 with differences by birth weight (2.6–<2500 gms; 10.2–≥2500 gms) and presentation (9.3 vaginal vertex, 10.1 Cesarean section vertex, 45.4 vaginal breech, 42.5 Cesarean breech). With increasing gestational, the incidence of neonatal hip instability increased for each category of gender and presentation (female vertex, female breech, male vertex, male breech).

### 3.4. Twin Studies and Genetics 

#### 3.4.1. Twin Studies

In 589 children with DDH, there were five pairs of twins (1.7%): two monozygotic and three dizygotic [162]. The frequency of concordance (both twins having the same pathology) was ~33% in monozygotic twins and ~8% in dizygotic twins [260] supporting a polygenic mechanism. Even with this high of a concordance rate, the majority of monozygotic twins are not concordant, demonstrating that the same genetic background and intrauterine environment does not result in DDH most of the time [260]. The Norwegian Twin Registry found an incidence of 4.3 DDH in twins (38 of 8794). The OR of DDH in a relative of an effected twin was 10.0 : 35.8 for mothers, 12.7 for siblings, 8.1 for fathers, and 3.3 for subsequent offspring [261]. The increased OR for mothers over fathers suggests a maternal effect. In 4678 opposite sex twins, the relative risk of DDH was 0.13 for males compared to females [262]. Mirroring has been described in two sets of identical twins [263, 264] with DDH; one twin had left DDH and the other right DDH. Both sets also had mirrored strabismus, and thumb sucking was mirrored in one set [263].

#### 3.4.2. Genetic Patterns

The prevalence of DDH in France in 1912 was 8 in Paris and 36–41 in Rennes, (Brittany region) [12, 184] with an epicenter in Pont l'Abbé. DDH did not occur in this geographic area in the 1700s, and it appeared that a genetic mutation occurred in the 19th century [184]. The genetic mechanism was postulated to be autosomal dominant with incomplete penetrance and penetrance dependent upon gender [12]. In 16 Turkish family members with DDH, an autosomal dominant inheritance was also noted [265]. 

Most other investigators propose a two-gene system in DDH. In 589 children with DDH [162], one gene involved joint laxity (dominant) and the other acetabular dysplasia (polygenic). The risk of subsequent members of a family having DDH when one family member had DDH was 36% when there was one affected parent and child, 12% when there was one affected parent and no affected children, and 6% when both parents were normal and had one affected child with DDH [266]. A Hungarian study [267] confirmed the role of polygenic acetabular morphometry and monogenic joint laxity; neonatal and “late-diagnosis” cases did not seem to be different entities. In an Italian study of pedigrees from 171 patients with familial DDH [268] (1937 individuals in 507 nuclear families with 499 having DDH), segregation analysis demonstrated that a two-locus recessive-recessive model was the most appropriate fit. The major locus demonstrated a penetrance of 5.9% in males and 58.6% in females, and the modifier locus 0.3% in males and 15.6% in females. 

Homozygous recessive conditions (attached earlobes, light eyes, color blindness, inability to roll the tongue, and 3 wrist creases) are increased in children with DDH [269]; 5.2 ± 0.1 in controls and 7.1 ± 0.2 in DDH.

#### 3.4.3. HLA and ABO Blood Type Groups

In Greek children with DDH [270] there was a significant increase in HLA A_1_ in the DDH group (52 versus 26%). In Czechoslovakia, there was a significant decrease in the HLA B_7_ type in the DDH group (10% versus 26%) [271]. In Japanese [272] the development of DDH and dysplastic osteoarthritis of the hip is associated with genes in the HLA-D regions, especially HLA DR4. No differences in ABO blood types [168, 259, 269, 273] or Rh blood types [259] have been found in DDH.

#### 3.4.4. Associated Genes and Chromosomal Locations

In Han Chinese, DDH was associated with gene growth differentiate factor 5 (CDF5) (OR 1.40) [274]. The double von Willebrand factor A gene on chromosome 3p24.3 is strongly associated with osteoarthritis of the knee in Japanese and Chinese [275] but not DDH [275]. Estrogen receptor Xba I wild type (XX compared to Xx and xx) is more common in DDH than controls (55.8% versus 37.9%) [276]. Homozygosity for the mutant Taq1 vitamin D receptor *t* allele is associated with an increased acetabular index, and the Pvu II pp estrogen receptor genotype is associated with a low center-edge angle [276]. 

Several studies associate DDH with chromosome 17q21. This region contains a cluster of HOX genes that provide specific positional identities to mesenchymal cells in developing joints [277]. In French Caucasians, there was no increased association between DDH and the HOXB9 gene [270] while in the Chinese there is an association with the HOXB9 as well as the *COL1A1* genes in DDH [278]. In Italian Caucasians, the *COLL2A1* and vitamin D receptor genes are associated with nonsyndromic DDH [279] but not confirmed by others [280]. In an 18 member multigenerational family affected by DDH, a 4 Mb region on chromosome 17q21.32 was linked to DDH [277].

### 3.5. Ligamentous Laxity

Joint laxity is increased in DDH [235, 266, 267, 281–283]: 75% of boys and 33% of girls with DDH demonstrate joint laxity [235]. The prevalence of joint laxity (≥3 joints) in normal children ages 6–11 years was 10.5% and 47% in DDH children [282]. Increased joint laxity likely explains the increase in inguinal hernias in DDH children and their fathers and brothers [142, 266, 283]. Girls with DDH have a 5-fold increase and boys with DDH a 3-fold increase in inguinal hernias; the hernia also develops earlier in life than normal [284]. Relaxin, which stimulates collagenase, alters the connective tissue and may lead to the development of both DDH and inguinal hernias. 

Collagen metabolism is altered in DDH. In newborns with DDH, the amount of umbilical cord collagen was decreased (~26%) [285] but not total protein [286]; this was not found in another study [173]. Umbilical cord type III/I collagen ratio is increased in DDH [286]. Hip capsule and skin biopsies in older DDH children (1 to 4 years old) show a decreased type III/I collagen ratio in the hip capsule but not the skin [287]. Opposite results were found in Japanese children with DDH [288] where the type III/I collagen ratio was increased in the hip capsule and ligamentum teres. The ligamentum teres in DDH children demonstrates thicker collagen fiber bundles and increased hyalinization in those with complete dislocation compared to subluxation [289]. In patients with hypermobility syndrome (including several cases of DDH) [290], growth hormone, insulin, and IGF-1 levels were elevated, leading us to endocrine abnormalities in DDH.

### 3.6. Hormonal/Endocrine and Metabolic Associations

DDH occurs predominantly in females, and a hormonal/endocrine relationship has long been suspected. In newborns with DDH, there is an increase in urinary excretion of conjugated estrogen and 17 *β*-estradiol [291–293], suggesting that DDH may be due to abnormal estrogen metabolism. Abnormal fetoplacental estrogen metabolism in a mother and child with DDH has been described [294]. 

Others found no estrogen metabolism abnormalities. Urinary estrogen excretion in the first 3 days of life in 7 newborns with bilateral Ortolani instability and 4 normal newborns was not different [295]. Urinary excretion of oestradiol, oestrone, and oestriol in 16 neonatal hip instability, and 19-matched-control newborns [296] showed no differences. Similar conclusions were reached by Borglin [297]. Umbilical cord blood measurements of 17 *β*-estradiol levels in 2185 consecutive newborns [298] with neonatal hip instability showed conflicting data; in boys low levels (<10 nmol/L) increased the risk of neonatal hip instability (OR 54) while in girls high levels (>15 nmol/L) increased the risk (OR 2.2). 

Women who develop pelvic joint instability and pain in pregnancy have an increased risk of a child with DDH [299]: incidence of 7–9 compared to the normal 2-3. In another study of Norwegian women with pregnancy-initiated pelvic pain, the incidence of DDH in the children was 45 [300], 7 times normal [179]. Women with pelvic joint instability and pain have higher serum relaxin levels compared to those without pelvic pain [301] in the third trimester which may explain these associations; others refute this [302, 303]. There is no correlation between serum relaxin in umbilical cord blood and neonatal hip instability [304, 305].

In adults previously treated for DDH without surgery, bone mineral density at the hip but not the spine is decreased with a concomitant increase in osteocalcin [306]. The OR of decreased bone mineral density at the hip was 6.3 for those with DDH. Some children with DDH demonstrate lower serum calcium and alkaline phosphatase but not serum phosphorus [307].

### 3.7. Seasonal Variation

Most studies note an increase in the number of DDH births in the winter. In Tokyo, 549 of 1276 (43%) DDH children were born on November–January [308]; in Kochi, Japan, 55 of 106 (52%) were born on December–February [81]. Other Japanese studies confirm this winter predominance [83, 90, 309], likely due to the increase of extension diapering/swaddling in the colder months. In Navajo boys, a slight increase was noted in the midwinter [37] but no seasonal variation was noted in other Navajos [40]. 

In Caucasians many studies also note a predominance of DDH births in the fall/winter months: September–December in Scandinavia [97–102]; September–November throughout Sweden [176, 310]; October–January in Israel [70]; November in Alicante, Spain [243]; September–January [311] and November–March in Leipzig [312]; December–February in Berlin [313], Northern Ireland [118], and Scotland [162]; December in all of the United States [231]; September–February in Utah [164]; September–March in England [110, 314, 315]; December-January in Debrecen, Hungary [141]; October–February in Budapest, Hungary [142]; November–February in Dêčín and Česká Lípa, Czechoslovakia [134]; December–March in Tel Aviv, Israel [201, 316]; and January–April in East Anglia, England [317]. In the southern hemisphere, the same was also noted: July-August in Southland, New Zealand [147], and in Australia, April–June in Victoria [318], June in Adelaide, and July in Brisbane [319]. Infants born in colder winter months demonstrate poorer acetabular development compared to those born in the warmer months [320] as measured by acetabular depth and acetabular angles; this may explain the increase in DDH in children born in the winter or may represent an effect of increased swaddling or tight clothing to protect the baby from the colder weather. Pelvic insufficiency also shows a seasonal variation [321] with a peak in November-December. 

Bimodal peaks were seen in Bardejov, Slovakia (July and February) [138]. Peaks were noted in the spring in Córdoba, Spain [121]; in the summer in Southampton, England [112], Helsinki, Finland [105], and Israel [202]. 

No seasonal variation was noted in Atlanta [322], Manchester and Newcastle-upon-Tyne, England [33, 114], Liberec, Czech Republic [136], Western Australia [44], Auckland, New Zealand [191], Christchurch and North Canterbury, New Zealand [146], and New York City [168].

### 3.8. Swaddling

Swaddling theoretically maintains the hip in an extended and adducted position; radiographs of hips in cradled infants demonstrate that they are not in true adduction, and up to 15–20° of abduction is possible [40]. Newborn swaddling used in many cultures [323] is considered by many to be a significant factor in the development of DDH. In ~2300 Navajos [40], hip dislocation was more prevalent in adults than children. It was postulated that this decrease in dislocation was due to the transition from exclusive use of the Navajo cradleboard to a more frequent use of diapers.

Swaddling/cradle boards in a few studies are not associated with DDH. In the Navajo, 9 of 13 cases of DDH which spontaneously improved showed improvement while on the cradleboard, and 2 cases with complete dislocation were never kept on the cradleboard [40]. Nearly all the Cree-Ojibwa swaddle their children (tihkinākan cradleboard) [38], yet in 427 untreated Cree-Ojibwa infants with hip dysplasia, frank improvement was seen in 113 with ≥1 year of followup. Most interestingly, 21 of the 113 had an initial diagnosis of complete dislocation [37]. Swaddling is also used in Saudia Arabia (mehad) [326] and Iran (ghondagh) [34] with no differences in the use of mehad/ghondagh for those with or without DDH. These studies question the role of swaddling. 

Most other studies demonstrate a strong correlation between swaddling and DDH. There was a 10-fold increase in DDH (123.0 versus 12.6) in Canadian Native Americans from Ontario who used the cradleboard [39]. Similar concerns were noted in the Cree from northern Quebec [327]. In Turkey [59], 98% of DDH cases were swaddled as infants compared to 87.1% not swaddled; the OR of DDH in swaddled children was 6.1 [223]. In a sonographic Turkish study [60], swaddling was used in 21.2% of those children with DDH ≥ Graf IIb (*P* < 0.001), which was the highest risk factor associated with DDH, greater than breech delivery (9.0%), positive family history (6.6%), and female gender (6.2%). The incidence of ultrasonographic dysplasia in children at high risk for DDH in Qatar [328] dropped from 20% to 6% after a community awareness program demonstrating the harmful effects of swaddling and discouraging its use; swaddling alone did not cause the hip dysplasia but had an unfavorable effect on the future course of a dysplastic hip. Another study from Saudi Arabia concluded that swaddling was an important factor in the etiology of DDH [329]. Swaddling is also believed to be responsible for the high incidence of DDH in the Arabic peoples in Western Galilee [61] and Iraqi immigrants in Israel [330].

**Figure 4:**
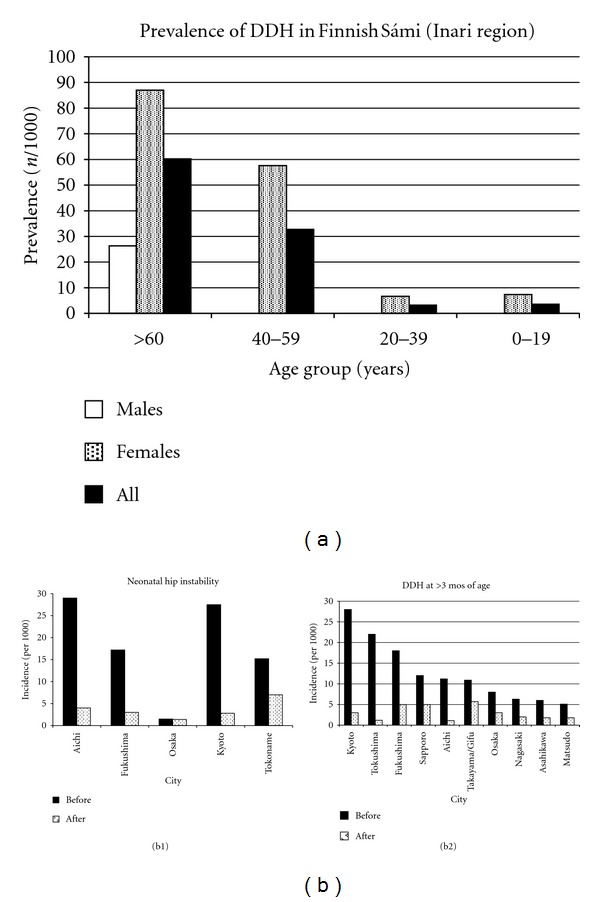
(a) The prevalence of DDH in the late 1970's by age group in the Sámi living in northern Finland, Lake Inari region. The overall prevalence was 16.3; for those ≥60 years of age it was 60.2, and dropped to 3.5 for those 0–19 years of age. This has been attributed to a decrease in the practice of newborn swaddling using the gietka or komse. Data from Eriksson et al. [324]. (b) The marked decrease in DDH incidence in Japan after introduction of a nationwide educational program for both neonatal hip instability and hip dislocation after 3 months of age. For neonatal hip instability the data was taken from [84, 325] and for hip dislocation from [83–90].

In Europe, Békés County, Hungary [139], swaddling was believed to account for the high incidence (28.7) of DDH and supported by others [134]. In the Swedish Sámi, the cradleboard (gietka or komse) was also believed to account for the high incidence (24.6) of DDH [46]. The gietka is a cradle hollowed from a log tightly swaddling the lower extremities and allowing for minimal movement. This cradle was very practical in the Sámi nomadic culture and lifestyle and allowed mothers to carry the cradle across their shoulders onto a reindeer's pack saddle; as their nomadic reindeer herding culture and lifestyle decreases and health nurses forbid the use of the geitka/komse [324, 331], the prevalence of DDH in the Sámi has fallen (Figure 4(a)). 

DDH is very rare in cultures where swaddling is not used (Southern Chinese, African Bantu, Thailand, North Korea, Sri Lanka [332]); the absence of swaddling is believed to be responsible for this [333]. Two different northern circumpolar peoples (Sámi and Inuit/Eskimos) have markedly different incidences of DDH [324, 334]; the Sámi, who swaddled their young in the past, had a very high incidence, while the Inuit/Eskimo's, whose mothers carried their young inside their parkas in a hood (amauti) abducting the hips around their backs have an incidence of DDH similar to Caucasians. 

In Japan, swaddling/diapering in extension was strongly associated with DDH [85]. This is especially so for children born in the winter months, being more tightly wrapped to protect against the cold [335]. This effect of swaddling/diapering with the hips in extension was initially noted in a small series of 5 normal newborn children; no hip instability was noted within the 1st 24 hours on physical examination, but after diapering in extension, 4 of 5 children developed Ortolani-positive hip instability [84]. This resulted in a larger study, and the creation of an educational campaign regarding the problems associated with extension diapering/swaddling in Kyoto, Japan [85]. The incidence of DDH dropped from 52.9 in 1971–97 to 5.6 in 1974–1976 after the educational campaign [85]. These results in Kyoto led to a national Japanese education and prevention campaign [335] with similarly striking results (Table 2(c), Figure 4(b)). This demonstrates the impact of epidemiological/demographic studies in reducing the occurrence of a particular condition.

Swaddling thus influences the development of DDH [336, 337]. This is particularly relevant since there is a strong resurgence to return to swaddling to reduce crying and promote uninterrupted sleep in the baby [336, 338, 339]. Swaddled infants arouse less and sleep longer; preterm infants show improved neuromuscular development, self-regulatory ability, and less physiologic distress when swaddled [338]. Infants that are at an increased risk of DDH should probably not be swaddled unless imaging studies are absolutely normal [336]; appropriate swaddling also must be used [340].

### 3.9. “Late Diagnosis” of DDH

When the hip actually dislocates [341–343] has been debated for some time [344]. The studies of “late” diagnosis reveal several findings. In one study, all the children were female, and 40% (8 of 20) had recognized risk factors [345]. Canadian children [346] diagnosed with DDH at an older age (20 months) have more right hip involvement (31% versus 15% right hips) compared to those diagnosed younger (<20 months) who have more left hip involvement (57% versus 15% left hips); bilateral dislocations are also more common when diagnosed at an older age (44 versus 28%) [346]. In Norway the incidence of “late” diagnosis was 2.4; of these 197 Norwegian children diagnosed “late” [347], all hips were stable at birth; 86% were female, the left hip was involved in 48%, right hip in 31%, and both in 21%. Breech delivery occurred in only 6.5%. The incidence of late DDH in all of Norway was 2.4 [347]: 1.7 in Oslo and 0.76 in southern Finland [348]. In Glasgow it was 0.84 before and 0.57 after institution of a selective ultrasound screening program [349]. A positive family history was associated with an increased risk of “late” DDH [350]. Avascular necrosis of the opposite normal hip in children with “late” diagnosis was described (9 of 103 cases) and associated with high, free-riding dislocations [351].

### 3.10. Miscellaneous Demographic and Epidemiologic Findings 

#### 3.10.1. In Utero Environment

**Exposures to Agents**

Administration of progesterone in the 1st trimester for threatened or habitual abortion [352] increases the risk of DDH as does maternal hypothyroidism [353] and maternal phenylketonuria [354]. Maternal smoking during pregnancy reduces the risk of sonographic DDH ≥ Graf IIa in female but not male neonates [355], most likely due to the effects of maternal smoke on estrogen physiology. The incidence of DDH in children whose mothers were exposed to radiation at Hiroshima or Nagasaki, Japan (0.31 and 0.23) is higher than those not exposed to radiation in Kure, Japan (0.13) [356]. In Seveso, Italy, no increase in DDH was seen after exposure of a densely populated area to TCDD (2,3,7,8-tetracholorobenzo-*p*-dioxin) [357]. Intrauterine toxoplasmosis [358] or viral infection [132] does not increase the risk of DDH. Iron deficiency anemia in fetal life may been associated with DDH [359].

**Mechanical and/or Physical Exposures**

Multifetal pregnancies (e.g., twins, triplets) are not at increased risk of DDH [142, 360–368]. There were no cases of DDH in 968 sets of twins and 18 sets of triplets in Tokyo [369]. Two studies noted less DDH in children born to multifetal pregnancies (relative risk 0.57 [370], 0.46 [371]). Although extrauterine pregnancy is rare (1 in 1100 to 1 in 50000 pregnancies) [372], it is often associated with DDH [373, 374], suggesting DDH is associated with molding forces in those circumstances rather than a teratological abnormality [372].

Mid-trimester amniocentesis does not increase the risk of DDH [375] whereas 1st trimester likely does [376] (OR 1.22). Premature labor and/or threatened abortion increases the risk of DDH [132, 377]. Prior miscarriage increases the risk of a subsequent child having DDH (OR 1.4) [378]. Preterm infants demonstrate a decrease in neonatal hip instability on physical examination compared to term infants [379] and do not demonstrate an increase in ≥Graf IIb DDH even though many are immature (Graf IIa) [380]. Preterm infants demonstrate no increase in DDH even after reaching normal development and gestational age [381].

#### 3.10.2. Parental Associations

DDH was more common in higher socioeconomic groups in Scotland [162] but more common in lower groups in Martin, Slovakia [137]; no differences in New York City [168] or England [110] were noted by socioeconomic class. Paternal plywood mill workers had an increased risk (OR 2.71) of DDH in 14415 British Columbian children [399].

Older paternal age has been associated with DDH [400]. Younger maternal age is a function of birth order and primiparity [164]. In one study, DDH and maternal age demonstrated a bell-shaped curve with a peak at 30 years of age [401]. Short maternal stature is associated with DDH in Canada [402] and Ireland [403] but not in Britain [403]. Early abduction treatment in children with neonatal instability is associated with increased maternal anxiety, while the use of ultrasound to manage infants with DDH is not [404].

#### 3.10.3. Child Development

The median walking age in 86 DDH children diagnosed at an average age of 16 months was not statistically different from a control group of 100 normal children with fractures (13.8 months—DDH; 12.5 months—control) [405]. Menarche in girls with DDH was decreased by 6 months compared to others [406]. DDH children have lower skin temperature on the affected lower limb [407].

### 3.11. Associated Conditions 

#### 3.11.1. Congenital Muscular Torticollis

DDH is associated with congenital muscular torticollis (CMT) [433–441]. The proportion of DDH in CMT children is 2.4% [441], 3.7% [434], 4.1% [437], 4.5% [440], 8% [435], 12% [438], 13% [439], 14.1% [442], 17% [443], and 20% [433]. Boys are particularly at risk [434]. CMT also has a higher incidence of breech presentation (19.5%) [437]. DDH risk is directly related to the severity of CMT [441]. The incidence of DDH in Hong Kong children with CMT is 48.1 compared to 0.1 in the general population [74].

#### 3.11.2. Congenital Foot Deformity

There is some association between DDH and metatarsus adductus [444–446]; the proportion of DDH in children with metatarsus adductus is 1.53% [444] and 10% [445]. The proportion of DDH children with congenital clubfoot is 0.3% (1 of 349) [447], 1% (16 of 1509) [446], and 5.9% (9 of 119) [448]. There is a slight association between positional clubfeet and DDH [447]. Acetabular dysplasia (acetabular index >28° at ≥4 months of age) occurred in 16% of children with congenital clubfoot [449]. Sonographic Graf type IV (irreducible DDH) occurred in 6.5% of children with congenital vertical talus and 4% with metatarsus adductus [450].

#### 3.11.3. Spinal and Neuromuscular Associations

Abnormal sensory evoked potentials in DDH children 10–13 months of age were present in 31% without spinal dysraphism and 56% with spina bifida occulta [451]. The incidence of DDH is 10 times greater in children with infantile scoliosis [452]. One study found no free nerve endings in either the ligamentum teres or hip capsule of DDH children [453] while the ligamentum teres had a higher frequency (66.6%) of type IVa free nerve endings [289] in DDH children, suggesting a role of abnormal nocioception/proprioception [454]. Vibration arthrometry demonstrates low-frequency vibrations in unstable (Ortolani- or Barlow-positive) neonatal hips [455, 456]. 

Electrophysiological and histological examinations of the hip muscles in DDH demonstrate muscle degeneration which is more severe in younger children [457]; the iliopsoas, gluteus medius, and vastus lateralis muscles demonstrated hyaline granulous degenerative changes [458]. Others note minimal histological changes [459].

#### 3.11.4. The Opposite Hip in Unilateral DDH

The opposite hip in “unilateral DDH” children often develops abnormally: 14% [460], 31% [461], 34% [462], and 43% [233]. The center-edge angle is the most predictive single measurement of delayed acetabular development while the overall best predictor is the hip ratio (CE angle + 100(acetabular depth/acetabular diameter))/2 [461]. A low ratio indicates a more abnormal hip; <22 is abnormal for those <2 years old and <23 for those ≥2 years old. This makes it possible to predict if the opposite hip is “at risk” [461]. Contralateral abduction hip contracture occurs in children with unilateral DDH [463, 464] as well as diffuse pelvic asymmetry if diagnosed after 4 months of age [465].

### 3.12. Transition from Childhood to Adult Hip Dysplasia

Persistent childhood dysplasia [466] and neonatal hip instability predisposes to adult hip disease. The Norwegian Medical Birth Registry was correlated with the Arthroplasty Registry [467]; when adjusting for gender and year of birth, there was a 2.6 times increased risk (95% CI 30–105) for children with neonatal hip instability to undergo total hip replacement. Of the 442 patients undergoing hip replacement, 95 had the surgery due to degenerative joint disease from residual hip dysplasia, yet only 8 had neonatal hip instability. This underscores the need for continued vigilance in the screening of neonatal hip instability and long-term followup. It also confirms that there is a significant amount of hip dysplasia, with no physical findings in childhood, that later becomes symptomatic in adult life (e.g., asymptomatic radiographic dysplasia). Underlying joint laxity may also predispose to the development of adult hip osteoarthritis [468, 469].

### 3.13. Adult Hip Dysplasia

The incidence and demographics/epidemiology of adult hip dysplasia is easier to study yet there are fewer and less detailed studies. The diagnosis of dysplasia, aside from complete dislocation, involves relatively simple radiographic measurements [470]. The center-edge (CE) angle of Wiberg is most commonly used to determine hip dysplasia [398] but only quantifies lateral coverage, ignoring other methods such as anterior coverage assessment with the false-profile radiograph [470]. The literature defining acetabular dysplasia is confusing; some studies use a CE angle <20°, while others use 22.5°, 25°, and 30° as the cutoff (Table 6). Also, intra- and interobserver agreement in CE angle measurement is ~±3% [471]. Considering these caveats, the prevalence of acetabular dysplasia ranges from 5.9% in Caucasians to ~19% in the Sámi.

Adult hip osteoarthritis (OA) is either primary or secondary. Primary OA is a diagnosis of exclusion and defined as when neither an anatomic abnormality nor any specific disease is identified as the cause [475]. Secondary OA is due to preexisting conditions, such as DDH, Perthes', and SCFE. Hoaglund [475] noted that normal hip anatomy was present in >80% of degenerative hip arthritis patients and that an underlying defect in articular cartilage or bone leads to the eventual development of OA in most Caucasians.

**Figure 5:**
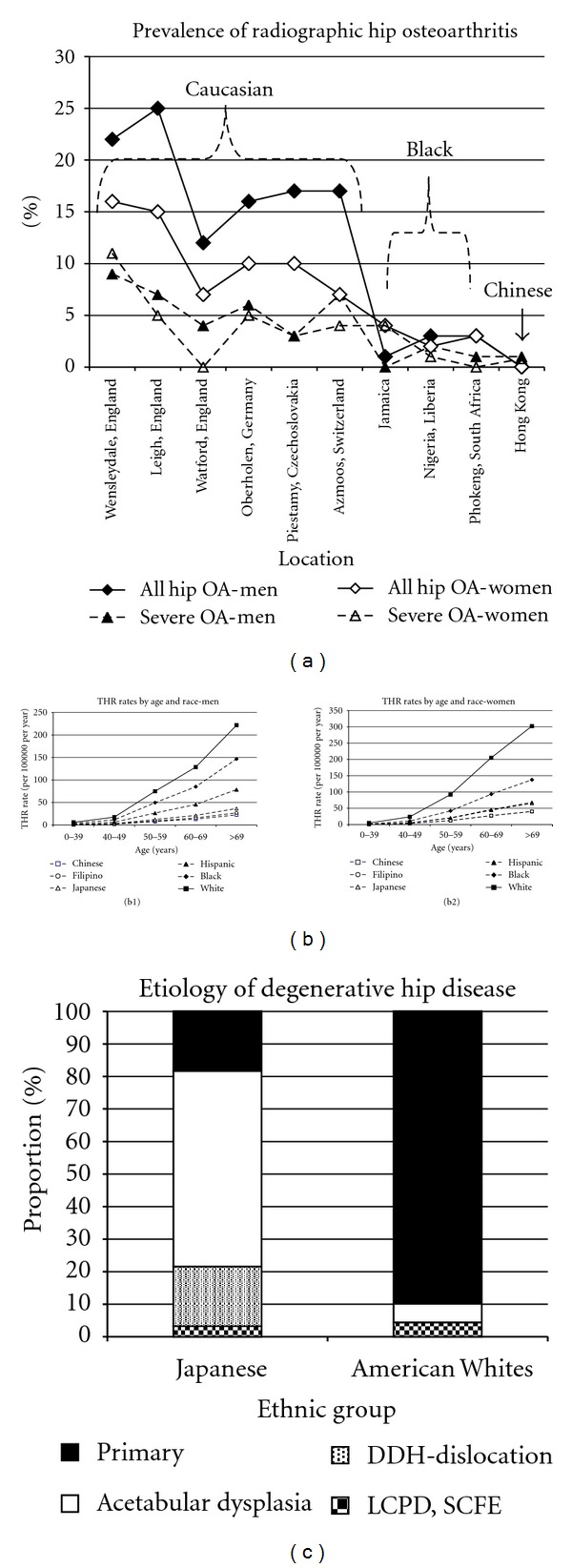
The demographics of hip osteoarthritis in adults. (a) Radiographic prevalence of hip osteoarthritis by ethnicity and geographic location. Data from Lau et al. [472]. (b1) Estimated rates of total hip replacements for primary coxarthrosis in San Francisco, 1984–1988, for men. Data from Hoaglund et al. [473]. (b2) Estimated rates of total hip replacements for primary coxarthrosis in San Francisco, 1984–1988, for women. Data from Hoaglund et al. [473]. (c) Differences in the etiology of adult hip arthritis between Japanese and Caucasian patients. Note the rarity of SCFE and Perthes' as a cause of adult hip arthritis. Data from Hoaglund et al. [474].

For the other 20%, what relations exist between mild dysplasia, ethnicity, and hip OA? The magnitude of adult acetabular dysplasia does not always correlate with degenerative hip disease in adults [385–387, 474, 475]. We must first know the prevalence of hip OA, and there are two ways to determine this number. One is the radiographic prevalence of hip OA. When using this method, the prevalence of radiographic hip OA is greater in men than in women and greater in Caucasians, intermediate in Blacks, and rare in Indo-Malays [472] (Figure 5(a)). The second is to determine total hip replacement rates, since truly symptomatic hip OA will likely come to THR. THR rates in San Francisco [473], in a population-based study, showed significant variation by ethnicity (Figure 5(b)), with Caucasians having a rate of THR ~10 times that of Indo-Malay peoples (Chinese, Japanese, Filipino), ~1.5 to 2 times that of Blacks, and ~3 to 4 times that of Hispanics. 

Variations in the frequency of hip OA are not necessarily caused by differences in the occurrence of hip dysplasia. There are marked differences in the etiology of hip osteoarthritis between Japanese Indo-Malays and American Caucasians [474]. In patients with osteoarthritis of the hip, secondary osteoarthritis was present in ~82% of the Japanese patients but only 10.1% of Caucasian Americans (Figure 5(c)). There is more acetabular dysplasia in Japanese adults but more hip OA in British Caucasians [385]. The prevalence of acetabular dysplasia (defined as a CE < 25°) was 0.5% in British Caucasians and 17.3% in Japanese, yet the prevalence of hip OA was lower in Japan (0% men, 2% women) compared to Britain (11% men, 4.8% women). In a random effects' statistical model, a negative relationship was actually discovered between acetabular dysplasia and joint space [385]; as acetabular depth increased (CE angle), joint space decreased. This was confirmed in Hong Kong where there is a lower prevalence of radiographic OA in Hong Kong men compared to British men, yet acetabular dysplasia was equal in prevalence in both groups [391]. It was also confirmed in France with a negative correlation between acetabular dysplasia and the risk of hip OA [390]. The highest prevalence of OA was in French men (5.8%) who had a low prevalence of hip dysplasia (1.8%), while the highest prevalence of acetabular dysplasia was in Japanese women (11.6%) who had a low prevalence of OA (3.5%). In Turkish adults [395] there is no direct correlation between acetabular dysplasia and hip OA. The prevalence of acetabular dysplasia is similar between British Caucasian men and Nigerian Black men, yet the prevalence of hip OA was less in the Nigerian men [382]. In the Sámi, there is no evidence that acetabular dysplasia is associated with OA [476].

In other populations; however, there is a definite correlation between acetabular dysplasia/residual childhood DDH and secondary hip OA [474, 477]. This is especially true in Asian populations [478], where the development of OA is nearly guaranteed if there is preexisting acetabular dysplasia or subluxation. In Japan, 97% of hip OA cases were secondary, with 88% due to DDH [479]. In elderly United States Caucasian women, mild acetabular dysplasia is associated with an increased risk of hip osteoarthritis [388]. The exceedingly low prevalence of acetabular dysplasia in Saudi people has been suggested as a contributing factor to their very low prevalence of OA [394].

### 3.14. Both Child and Adult Hip Dysplasia—Archeological Evidence

There has been significant interest in hip dysplasia and archeological remains (Table 7). A systematic review in this domain is quite difficult due to the many detailed, but hard to identify and access, theses and monographs. Thus, the papers discussed here are not included in the 422 of 2277 manuscripts reviewed. We attempted to review the literature as best as possible, well recognizing that some studies have been missed. Many archeological studies are not applicable being solely a case description of a skeleton with DDH with no data from which a prevalence can be calculated. In archeological studies, prevalence rather than incidence is calculated. As DDH begins some time in infancy, and since there were no diagnostic/treatment methods for children with DDH in the eras of these archeological studies, prevalence and incidence become identical. With anthropological materials, the exact diagnosis may be questioned due to many different types of hip dislocation. However, a critical review was performed, and all attempts were made to exclude posttraumatic, neuromuscular, or infectious dislocations.

#### 3.14.1. Europeans

In 700 skeletons buried in six medieval (4th to 13th century) sites from Northern and Eastern France [408], six had DDH. In 900 skeletons from the Notre-Dame-du-Borg cathedral gravesite (8th to 17th century) in Southern France (Digne) [409], nine had DDH. In the Spitalfields cemetery in London, used between 1150 and 1530 AD [410], 3290 pairs of hips were sufficiently preserved for study; nine had DDH. In 100 skeletons (all affluent citizens) from a Minorite monastery churchyard cemetery in Dordrecht, Netherlands, one had DDH [414]. In 70 skeletons from post-medieval (1550 to 1700) Bobald, Transylvania, Romania [412], two had DDH. In327 skeletons at Devín, Slovakia (9th–12th century AD) [413], DDH was noted in one. In 271 skeletons from the Southern East Siberian area of Cis-Baikal (6800–1000 BC) one had DDH [415]. 

In the Mediterranean areas, a study of 139 Herculaneum people buried by the Mt. Vesuvius volcano in 79 AD demonstrated one DDH [418]. In Padua, Italy, a study of 213 skeletons (Tedeschi Osteological Collection) from the late 19th and early 20th centuries demonstrated one DDH [417]. In a study of 225 skeletons from Southern Greece in the 6th-7th centuries AD there were no cases of DDH [416].

#### 3.14.2. Mid-Eastern/Egyptians

In the early Bronze Age (3150–2200 BC) people of Bâb edh-Dhrâ, Jordan, there was one case of DDH in 92 skeletons [420]. None of 73 Bedouin skeletons (200 BC) from Tel Sheva, Israel, demonstrated DDH [419]. In 24 skeletons from Gisr el-Mudir, Saqqara, Egypt during the 2nd Dynasty (2890–2650 BC), there was one case of DDH [421].

#### 3.14.3. Native Americans

The first natives encountered by Christopher Columbus were the Taino people [480]. In 102 Taino skeletons dated from the late 15th century found at Juan Dolio, Maguana, Santo Domingo, Dominican Republic [432], 5 cases of DDH were noted. In 100 Mound Builder peoples' skeletons along the Mississippi River in eastern Arkansas [425], (precolonization era, before 1500), there was one unilateral case of DDH. In 75 Caddo-Indian peoples who lived in what is now the Red River county, Eastern Texas (1100–1800 AD) [429], there was one DDH. In the Ontario Uxbridge Iroquoian ossuary (~1490 AD), one DDH was seen in 312 skeletons [423]. In 286 skeletons from the Fort Erie, Niagara River, Ontario (precolonization era), two cases of DDH were noted [424]. In 486 Native American Crow Creek villagers massacred in the 14th century on the east bank of the Missouri River in South Dakota [426], one DDH was found (a 6–10 years old child). In 54 skeletons from the Kechipawan Pueblo, New Mexico (1300–1600 AD), no DDH was seen [422]. In two small series of 26 skeletons each from El Morro Valley, New Mexico (13th century) [430] and Sundown, Prescott, Arizona (1100–1200 AD) [431], there were no cases of DDH.

The overall gender and laterality mix in the archeological samples is very similar to the composite results in this paper (Table 7). In those skeletons with DDH where the data was known, 74% were female and 35% demonstrated bilateral involvement, similar to the figures of 76% female and 27% bilateral in this paper. The prevalence of DDH in these archeological samples, even though most are small in size except for that from the Spitalfields cemetery, demonstrates differences compared to the incidence of DDH in this paper. Those for Western Europe are similar (4.9 archeological, 3.6 present United Kingdom). All others were different: Eastern Europe (6.0 archeological, 35.8 present), Mediterranean (3.5 archeological, 25.5 present), Middle East (10.6 archeological, 6.2 present), and indigenous Native Americans (6.1 archeological, 76.1 present). If the sampling of the archeological data set was adequate, then statistical analysis should determine if such differences were real. We subjected the data to the Fisher two-tailed exact test, analyzing for differences between the archeological data and the present data. These data (DDH *n*/total *n*) and (archeological versus present) were 25/5089 and 3232/906428 for Western Europe; 4/668 and 12452/348031 for Eastern Europe; 2/577 and 2240/87581 for Mediterranean; 2/189 and 732/118225 for Arabic Middle East; 44/8321 and 1108/14553 for indigenous Native Americans. There were no differences for Western Europe (*P* = 0.12) and the Middle East Arabic (*P* = 0.33), while there were differences for Eastern Europe and indigenous Native Americans (*P* < 0.001). These differences may represent changing epidemiology, changes in natural selection processes in the archeological era, or inadequate sampling of the archeological materials. Further inquiry will be needed in this subject area.

## 4. Conclusion

DDH demonstrates a predominance of left-sided (64.0%) and unilateral involvement (63.4%). The incidence per 1000 live births ranges from 0.06 in Africans in Africa to 76.1 in Native Americans with significant variability between and within racial groups and geographic location. The incidence of clinical neonatal hip instability at birth ranges from 0.4 in Africans to 61.7 in Polish Caucasians. Predictors of DDH are breech presentation, positive family history, and gender (female). Ligamentous laxity and abnormalities in collagen metabolism, estrogen metabolism, and pregnancy-associated pelvic instability are well-described associations with DDH. Many studies demonstrate an increase of DDH in the winter, both in the Northern and Southern hemispheres. Swaddling is strongly associated with DDH. Associated conditions are congenital muscular torticollis and congenital foot deformities. The opposite hip is frequently abnormal when using rigorous radiographic assessments. Archeological studies demonstrate that the epidemiology of DDH may be changing.

## Figures and Tables

**Table 1:** Graf's classification of hip dysplasia using ultrasound.

Graf's hip type	Description	*α* angle (°)	*β* angle (°)	Age
I	Normal	>60	<55	Any
IIa	Physiologically immature	50–60	55–77	0–12 wks
IIb	Immature	50–59	55–77	>12 wks
IIc	Acetabular deficiency	43–49	>77	Any
IId	Everted labrum with subluxation	43–49	>77	Any
III	Everted labrum with dislocation	<43	>77	Any
IV	Dislocation	<43	>77	Any

As described by Graf [23], Roposch et al. [24], and Herring [25].

**Table:** (a) Indigenous peoples

Study	Year	Location	Ethnicity	Dx	No. Pts	No. DDH	Incidence (per 1000)
***Native Americans***							
Corrigan and Segal [36]	1950	Island Lake, Manitoba	Cree-Ojibwa	Documented DDH	1253	45	35.9
Walker [37]	1977	Island Lake, Manitoba	Cree-Ojibwa	All DDH	1248	420	336.5
				Dislocation		243	194.7
				Dysplasia		123	98.6
				Other		54	43.3
Houston and Buhr [38]	1966	Northern Saskatchewan	Cree	All DDH	4453	59	13.2
				Likely DDH	1253	71	56.7
Salter [39]	1968	Ontario, Canada	—	All DDH			
				Used cradleboard	2032	250	123.0
				No cradleboard	1347	17	12.6
Rabin et al. [40]	1965	Many Farms District, AZ	Navajo	*Adults—All*	270	9	33.3
				Dislocation		7	25.9
				Dysplasia		2	7.4
				*Children—All*	548	22	40.1
				Dislocation		4	7.3
				Dysplasia		18	32.8
				*Adults and Children—All*	818	31	37.9
				Dislocation		11	13.4
				Dysplasia		20	24.4
Pratt et al. [41]	1982	Many Farms District, AZ	Navajo	*Children*			
				All DDH	548	18	32.8
				Dislocation		14	25.5
				Dysplasia		4	7.3
				*Adults*			
				All DDH	270	89	330
				Dislocation		70	259
				Dysplasia		19	70
Coleman [42]	1968	Fort Defiance, Ship Rock, Gallup	Navajo	All DDH ≤ 3 months old	1155	77	66.7
Kraus and Schwartzman [43]	1957	Fort Apache	Apache	Dislocation	3500	107	30.6
*Weighted avg.*				All DDH	14553	1108	76.1
***Sámi and Australian Aboriginals***							
Bower et al. [44]	1987	Western Australia	Australian Aboriginals	All DDH	*	22	37
Getz [45]	1955	Sámpi (Circumpolar Europe)	Sámi	All DDH	*		40
Mellbin [46]	1962	Sweden	Sámi	All DDH	813	20	24.6

**Table:** (b) Africans, Indo-Mediterranean, and mixed peoples

Study	Year	Location	Ethnicity	No. Pts	No. DDH	Incidence (per 1000)
***Africans—Blacks***						
Edelsetin [47]	1966	South Africa	Bantu	16678	0	0
Roper [48]	1976	Rhodesia (Zimbabwe)	Bantu	40000	1	0.025
Pompe van Meerdervoort [49]	1977	South Africa	—	10000	3	0.3
*Weighted avg.*				66678	4	0.06
Burke et al. [50]	1985	United States	—	28261*	13	0.46
Finley et al. [51]	1994	Jefferson County, Alabama, USA	—	9654	2	0.2
*Weighted avg.*				37915	15	0.40
***Indo- Mediterranean***						
Kulshrestha et al. [52]	1983	Ballabhgarh, India	Indian	2409	1	0.42
Singh and Sharma [53]	1980	New Delhi, India	Indian	7274	7	1.0
Boo and Rajaram [54]	1984	Kuala Lumpur	Indian	8109	10	1.23
Gupta et al. [55]	1992	New Delhi, India	Indian	6209	16	2.65
Ang et al. [56]	1997	Singapore	Indian	2810	13	4.6
Kaushal et al. [57]	1976	Chandigarh, Northern India	Indian	2500	23	9.2
Şahin et al. [58]	2004	Ankara, Turkey	Turkish	5798	10	1.7
Kutlu et al. [59]	1992	Konya, Turkey	Turkish	4173	56	13.4
Doğruel [60]	2008	Ankara, Turkey	Turkish	3541	167	47.2
Alkalay [61]	1972	Tamra, Galilee, Israel	Arabic	450	21	46.7
Alkalay [61]	1972	Western Galilee, Israel	Arabic/Druze	3625	109	30.0
Moosa et al. [62]	2009	Dubai, UAE	Arabic	3786	12	3.17
Mirdad [63]	2002	Aseer, Saudi Arabia	Saudi	79548	300	3.8
Danielsson [64]	2000	Malmö, Sweden	Iraqi/Iranian	1604	7	4.4
Mamouri et al. [65]	2004	Mashhad, Iran	Iranian	6576	10	1.5
Abdinejad et al. [34]	1996	Shiraz, Iran	Iranian	8024	30	3.6
Pashapour and Golmaham- madlou [66]	2007	Urmia, Iran	Iranian	1100	10	9.1
Paterson [67]	1976	Western Australia	Indo-Mediterra- nean, not otherwise specified	2964	9	3.0
*Weighted avg.*			*All*	150500	811	5.4
			*Indian*	29311	70	2.4
			*Arabic*	118225	732	6.2
***Mixed/ Unknown—All Geographic Locations***						
Rao and Thurston [68]	1986	Wellington, New Zealand	Not specified	15174	60	4.0
Lowry et al. [69]	1989	Alberta, Canada	North America	813^@^	30347	2.68
			Not specified	34956	342	9.8
Medalie et al. [70]	1966	Jerusalem, Israel	Dislocation		107	3.1
			Subluxation		235	6.7
Harlap et al. [71]	1971	Jerusalem, Israel	Jewish/Arabic	18017	104	5.7

^@^calculated from the given incidence and total number of births.

**Table:** (c) Indo-Malay peoples

Study	Year	Location	Ethnicity	No. Pts	No. DDH	Incidence (per 1000)
Huang et al. [72]	1988	Taiwan	Chinese	9884	10	1.01
Chang et al. [73]	2007	Taiwan	Chinese	*	*	2.9
Hoaglund et al. [74]	1981	Hong Kong	Chinese	557683	38	0.07
Boo and Rajaram [54]	1984	Kuala Lumpur	Chinese	12115	4	0.33
Limpaphayom [75, 76]	1975	Thailand	Thai	33433	17	0.5
Ang et al. [56]	1997	Singapore	Malay	7439	40	5.4
Boo and Rajaram [54]	1984	Kuala Lumpur	Malay	29695	21	0.71
***Japanese—before Educational/ Prevention Campaigns***						
Naito [77]	1958	Japan	Japanese	*	*	56.0
Akabayashi [78]	1958	Miyagi, Japan	Japanese			33.0
Tsuji [79]	1964	Tokyo, Japan	Japanese			11.9
Kashiwagi and Kagawa [80]	1965	Kobe, Japan	Japanese	929	41	44.1
Haginomori [81]	1966	Kochi, Japan	Japanese	3323	106	31.9
Tanabe et al. [82]	1972	Okayama, Japan	Japanese			
			All	2756	73	26.5
			Dislocation		32	11.6
			Subluxation		41	14.9
Wada et al. [83]	1993	Tokushima Prefecture,	Japanese			22*
Ishida [84]	1993	Aichi	Japanese			11.2*
Ishida [84]	1993	Fukushima	Japanese			18*
Ishida [84]	1993	Osaka	Japanese			8*
Ishida [85]	1993	Kyoto	Japanese			28*
Kikuike et al. [86]	1993	Takayama/Gifu	Japanese	2289	25	10.9
Gotoh et al. [87]	1993	Asahikawa	Japanese	15944	95	6
Saito [88]	1993	Sapporo	Japanese			12*
Shinohara [89]	1993	Matsudo	Japanese			5.1*
Iwasaki and Takahashi [90]	1993	Nagasaki	Japanese			6.3*
***Japanese—Seminal Study on Effects of Extension Diapering/Swaddling***						
			Japanese			
Ishida [85]	1977	Kyoto, Japan	Swaddled	3778	200	52.9
			Not swaddled	3047	17	5.6
***Japanese— after Educational/Prevention Campaigns***						
Higuchi [91]	1984	Tokyo and Ibaragi Prefecture	Japanese	13379	45	3.4
Wada et al. [83]	1993	Tokushima Prefecture,	Japanese	17224	20	1.2
Ishida [84]	1993	Aichi	Japanese			1.1*
Ishida [84]	1993	Fukushima	Japanese			5*
Ishida [84]	1993	Osaka	Japanese			3*
Ishida [85]	1993	Kyoto	Japanese			3*
Kikuike et al. [86]	1993	Takayama/Gifu	Japanese	1749	10	5.7
Gotoh et al. [87]	1993	Asahikawa	Japanese	9471	17	1.8
Saito [88]	1993	Sapporo	Japanese			5.0*
Shinohara [89]	1993	Matsudo	Japanese			1.8*
Iwasaki and Takahashi [90]	1993	Nagasaki	Japanese			2.0*
			*All*	714254	769	1.08
			*Chinese*	57962	52	0.1
*Weighted avg.*			*Malay*	37134	61	1.6
			*Japanese*			
			* Before*	25241	340	13.5
			* After*	41823	92	2.2

^∧^Incidence from [82].

*Only the incidence was given and could not be included in the weighted averages.

**Table:** (d) Caucasians

Study	Year	Location	No. Pts	No. DDH	Incidence (per 1000)
***Scandinavia***					
Severin [92]	1956	All Sweden	566142	497	0.88
von Rosen [93]	1962	Malmö, Sweden	24000	40	1.7
von Rosen [94]	1968	Malmö, Sweden	31304	171	5.46
Fredensborg [95]	1976	Malmö, Sweden	58579	548	9.33
Danielsson [64]	2000	Malmö, Sweden	15189	115	7.57
Beckman et al. [96]	1977	Northern Sweden	40419	295	7.30
Finley et al. [51]	1984	Uppsala, Sweden	62879*	193	28.0
Bjerkeim [97–102]	1974, 1976	Southeastern Norway	*	*	10.0
Finne et al. [103]	2008	Oslo, Norway	19820	34	1.7
Melve and Skjaerven [104]	2008	All Norway	519266	2509	4.83
Heikkilä [105]	1984	Southern Finland	151924	1035	6.81
Clausen and Nielsen [106]	1988	Randers, Denmark	13589	83	6.1
*Weighted avg.*			1510007	5713	3.8
***Western Europe***					
Mitchell [107]	1972	Edinburgh, Scotland	31961	100	3.1
MacKenzie and Wilson [108]	1981	Aberdeen, Scotland	53033	1606	30.3
Bertol et al. [109]	1982	Edinburgh, Scotland	44953	299	6.7
Record and Edwards [110]	1958	Birmingham, England	226038	148	0.66
Leck et al. [111]	1968	Birmingham, England	94474	86	0.91
Wilkinson [112]	1972	Southampton, England	6272	37	5.9
Jones [113]	1977	Hertfordshire, England	29366	76	2.6
Noble et al. [114]	1978	Newcastle upon Tyne, England	25921	271	10.5
Catford et al. [115]	1982	Southampton, England	76724	178	2.32
Knox et al. [116]	1987	Birmingham, England	144246	96	0.67
Williamson [117]	1972	Northern Ireland	34840	97	2.78
Patterson et al. [118]	1995	Belfast, Northern Ireland	138600	243	1.75
Reerink [119]	1993	Leiden, Netherlands	2092	32	15.3
Judet and Tanzy [120]	1966	Creuse, France (only girls)	1326	48	3.6
Valdivieso Garcia et al. [121]	1989	Córdoba, Spain	33000	323	9.79
Padilla-Esteban et al. [122]	1990	Madrid, Spain	40243		
		All		1747	43.4
		Dislocation		89	2.21
		Subluxation		80	1.99
		Dysplasia		1587	39.4
Sanz et al. [123]	1991	Salamanca, Spain	6135	54	8.8
Giannakopoulou et al. [124]	2002	Crete	6140	65	10.6
Di Bella et al. [125]	1997	Sicily	2000	51	25.5
		*All*	996038	5509	5.5
*Weighted avg.*		*United Kingdom*	906428	3232	3.6
		*Mediterranean/ Spain*	87518	2240	25.5
***Eastern Europe***					
Srakar [126]	1986	Ljubljana, Yugoslavia	5000	50^@^	10.0
Kepeski et al. [127]	1969	Skoplje, Macedonia	9149	302^@^	33.0
Maričević [128]	1885–1993	Lastovo Island, Croatia	3676	19	5.2
Krolo et al. [129]	1968–88	Zagreb, Croatia	7168	120	16.7
Stipanicev [130]	1985	Šibenik, Croatia	26227	2203^@^	84.0
Darmonov [131]	1996	Stara Zagora, Bulgaria	20417	124	6.1
Samborska and Lembrych [132]	1973	Opole, Poland	14500	159	11.0
Polívka [133]	1973	West Bohemia, Czech Republic	28471	3223	113.2
Košek [134]	1973	Dêčín and Česká Lípa, Czech Republic	23580	1048	44.4
Poul et al. [135]	1992	Brno, Czechoslovakia	35550	656	18.5
Vencálková and Janata [136]	2009	Liberec, Czech Republic	12944	335	25.9
Drimal [137]	1959	Martin, Slovakia	9510	120	12.6
Tomáš [138]	1989	Bardejov, Slovakia	7208	323	44.8
Czéizel et al. [139]	1974	Békéscsaba, Hungary	18219	523	28.7
Csató and Benkó[140]	1963	Miskole, Hungary	5513	30	5.44
Pap [141]	1956	Debrecen, Hungary	11933	217	18.2
Czeizel et al. [142]	1972	Budapest, Hungary	108966	3000	27.5
*Weighted avg.*			348031	12452	35.8
***Australia and New Zealand***					
Paterson [67]	1976	South Australia	4445	31	7.0
Yiv et al. [143]	1977	South Australia	19622	206	10.5
Bower et al. [44]	1987	Western Australia	62879^∧^	415	6.6
Chan et al. [144]	1999	Adelaide, Australia	118379	916	7.74
Howie and Phillips [145]	1970	Auckland, New Zealand	16103	57	3.54
Doig and Shannon [146]	1975	Canterbury, New Zealand	23443	62	2.65
Dykes [147]	1975	Southland, New Zealand	47064	103	2.19
Hadlow [32]	1988	New Plymouth, New Zealand	20657	331	16.0
*Weighted avg.*			312592	2121	6.8
***Americas***					
Lehmann and Street [148]	1981	Vancouver, British, Columbia, Canada	116808	142	1.2*
Tijmes et al. [149]	1971	Llanquihue, Chile	30000	137	4.6
Hazel and Beals [150]	1989	Portland, Oregon	39429	32	0.8
Finley et al. [51]	1994	Jefferson County, Alabama	17907	12	0.7
*Weighted avg.*			174144	186	1.07

*The incidence and either the numerator/denominator were given; appropriate values calculated when possible.

^@^calculated from the given incidence and total number of births.

**Table 3:** Incidence of neonatal hip instability by screening physical examination.

Study	Year	Location	No. Pts	No. DDH	Incidence (per 1000)
*Africans*					
Robinson and Buse [166]	1979	Kampala, Uganda	2000	4	2.0
Gross et al. [167]	1982	Oklahoma City, Oklahoma	2686	1	0.4
Artz et al. [168]	1975	New York City	4286	18	0.42
*Indo-Mediterranean*					
Abdel-Kader and Booz [169]	1968	Kuwait	4000	5	1.25
Al-Umran et al. [170]	1988	Dammam, Saudi Arabia	12733	62	4.9
Khan and Benjamin [171]	1992	Abha, Saudi Arabia	2222	81	36.5
*Amerindian*					
Hernández-Arriaga et al. [172]	1991	Guanajuato, México	16987	25	1.47
*Indo-Malay*					
Morito [173]	1983	Okayama, Japan	4824*	51*	10.6
Chen [174]	1967	Taipei, Taiwan	2257	4	1.8
Hsieh et al. [175]	2000	Taichung, Taiwan	3345	4	1.2
*Caucasians—Scandinavia*					
Andrén [176]	1962	Malmö, Sweden	28292	64	2.26
von Rosen [177]	1970	Malmö, Sweden	34520	171	4.94
Palmén [178]	1961	Falköping, Sweden	12394	70	5.65
Hinderaker et al. [179]	1994	All Norway	959412	9483	9.88
Beckman et al. [96]	1970–73	Västerbotten County, Sweden	11613	119	10.2
Almby and Rehnberg [180]	1977	Uppsala, Sweden	29339	298	10.2
Hiertonn and James [181]	1968	Uppsala, Sweden	11868	242	20.4
Medbö [182]	1961	Ålesund, Norway	3242	50	15.4
Cyvín [183]	1977	Trondheim, Norway	6509	146	22.4
*Caucasians—Western Europe*					
Dickson [184]	1912	Paris, France	1502	12	8.0
		Rennes, France	220	9	41.0
Jones [113]	1977	Norwich, England	29366	76	2.58
Finlay et al. [161]	1967	Uxbridge, England	14594	60	4.1
O'Brien and McGill [185]	1970	Dublin, Ireland	10081	77	7.6
Barlow [33]	1962	Salford, England	9289	139	14.9
Wilkinson [112]	1972	Southampton, England	6272	37	5.9
Galasko et al. [186]	1980	Salford, England	11980	179	14.9
Dunn et al. [187]	1985	Bristol, England	23002	445	19.3
Lennox et al. [188]	1993	Aberdeen, Scotland	67093	3354	50.0
Mitchell [107]	1972	Edinburgh, Scotland	31961	226	7.1
Drescher [189]	1957	Leipzig, Germany			
		All	5098	164	32.2
		Vertex	4953	104	30.0
		Breech	145	19	131
*Caucasians—Eastern Europe*					
Szulc [190]	1961–66	Poland	2608	161	61.7
*Caucasians—Australia and New Zealand*					
Phillips [191]	1968	Auckland, New Zealand	43025	148	3.4
Bower et al. [192]	1989	Western Australia	67757	450	6.6
Chaitow and Lillystone [193]	1984	Sydney, Australia	450	3	6.7
Goss [194]	2002	Victoria, Australia	5166	100	19.4
*Caucasians—North America*					
Coleman [195]	1956	Salt Lake City, Utah	3500	30	8.6
Ponseti [196]	1978	Iowa City, Iowa	51359	72	1.4
Gross et al. [167]	1982	Oklahoma City, Oklahoma	7490	39	5.2
Lehmann and Street [148]	1981	Vancouver, British Columbia, Canada	23234	132	5.7*
Tredwell and Bell [197]	1981	Vancouver, British Columbia, Canada	32480	321	9.9
Ritter [198]	1973	Indianapolis, Indiana	3278	30	9.2
Artz et al. [168]	1975	New York, New York	19020	291	15.3
		*All*	1528069	16452	10.8
		*Scandinavia*	1085576	10524	9.7
*Caucasian's weighted avg.*		*Australia/New Zealand*	116398	701	6.0
		*Western Europe*	185734	4312	23.2
		*North America*	140361	915	6.5
*Mixed/Unknown—All Geographic Locations*					
Ein [199]	1957	Newark, New Jersey	4597	7	1.5
Stanisavljevic [200]	1962	Detroit, Michigan	5125	35	6.8
Weissman and Salama [201]	1969	Tel Aviv, Israel	6841	45	2.7
					
Klingberg et al. [202]	1976	Rehovot, Israel			
			12150	172	14.2
			6204	49	7.9
			5946	123	20.7
Khrouf et al. [203]	1986	Tunis, Tunisia	10000	41	4.1

*The incidence and either the numerator/denominator were given; appropriate values calculated when possible.

**Table 4:** Incidence of DDH in the ultrasound screening period era (1980s–present)*.

Study	Year	Location	Ethnicity	Time	No. Pts	No. DDH	Incid.	> Graf IIa	Incid. > Graf IIa
*At birth—2 weeks*									
Eidelman et al. [157]	2002	Ethiopia	Black Jews	Birth	768	19	24.7	10	13.0
Poul et al. [208]	1998	London, England	Black	Birth	185	0	0.0	0	0.0
Chang et al. [73]	2007	Taiwan	Indo-Malay (Chinese)						
Danielsson [64]	2000	Malmö, Sweden	Indo-Med. (Iraqi/Iranian)		1604	7	4.4		
Danielsson [64]	2000	Malmö, Sweden	Caucasian	Birth	15189	115	7.6		
Treiber et al. [209]	2008	Maribor, Slovenia	Caucasian	Birth	17393	324	18.6	369	21.2
Vencálková and Janata [136]	2009	Liberec, Czech Republic	Caucasian	Birth	16678			212	12.7
Rosendahl et al. [210]	1996	Bergen, Norway	Caucasian	Birth	3613	1613	446.4	123	34.0
Bache et al. [211]	2002	Coventry, England	Caucasian	Birth	29323	3866	131.8	2340	79.8
Szöke et al. [212]	1988	Cologne, Germany	Caucasian	Birth	1000	524	524.0	40	40.0
Tönnis et al. [213]	1990	Dortmund, Germany	Caucasian	Birth	2587	1877	725.6	137	53.0
Rühmann et al. [214]	1998	Hanover, Germany	Caucasian	Birth	6617	436	65.9	217	32.8
Parten- heimer et al. [215]	2006	Greifswald, Germany	Caucasian	4–10 days	2256			110	48.8
Exner [216]	1988	Zurich, Switzerland	Caucasian	Birth	615	521	847.2	28	45.5
Peled et al. [217]	2008	Haifa, Israel	Caucasian	Birth	45497			2137	47.0
Giannako- poulou et al. [124]	2002	Crete	Caucasian	2 wks	6140	65	10.6	50	8.1
Ballerini et al. [218]	1990	Milan, Italy	Caucasian	Birth	2842	778	273.8	57	20.1
Riboni et al. [219]	1991	Milan, Italy	Caucasian	Birth	1507	508	337.1	15	10.0
Franchin et al. [220]	1992	Bari, Italy	Caucasian	Birth	3000	959	319.7	309	103.0
Baronciani et al. [20]	1997	Lecco, Italy	Caucasian	Birth	4648	1186	255.2	267	57.4
Riboni et al. [221]	2003	Milan, Italy	Caucasian	Birth	8896	2008	225.7	34	3.8
Yiv et al. [143]	1997	South Australia	Caucasian		19622	206	10.5		
*Weighted average (Caucasians)*					187423	14986	80.0	6445	42.2
*At 4 to 6 weeks*									
Eidelman et al. [157]	2002	Ethiopia	Black Jews	6 wks	768	3	3.9	3	3.9
Doğruel et al. [60]	2008	Ankara, Turkey	Indo-Med (Turkish)	6 wks	3541	167	47.2	208	58.7
Bache et al. [211]	2002	Coventry, England	Caucasian	6 wks	29323	92	3.1		
Roovers et al. [222]	2005	Enschede, Netherlands	Caucasian	4 wks	4473	1697	379.4	132	29.5
*Weighted average (Caucasians)*					33796	1789	52.9	132	29.5
*At 4 to 6 Months*									
Krolo et al. [129]	1989–2001	Zagreb, Croatia	Caucasian	4 months	2010	120	59.7	15	7.5
Akman et al. [223]	2007	Ankara, Turkey		6 months	403			14	34.7

*The data for the last two columns those having > Graf IIa instability are for all hips, while the previous columns are for children.

**Table 5:** General demographics of childhood hip dysplasia.

Study	Year	Location	Ethnicity	No. DDH	M	%M	F	%F	B	%Bil.	U	%Unil.	R	%R	L	%L	%RU	%LU
Ang et al. [56]	1997	Singapore	Indo-Malay (mixed)	96	35	37	61	63										
Chai and Sivanan- tham [224]	1990	Kuala Lumpur, Malaysia	Indo-Malay (mixed)	22	5	23	17	77	3	14	19	86	11	58	8	42		
Wada et al. [83]	1993	Tokushima, Japan	Indo-Malay (Japanese)	20	2	10	18	90	4	20	16	80	7	29	17	71	19	81
Kaushal et al. [57]	1976	Chandigarh, India	Indo-Med (Indian)	23	19	83	4	17					9	39	14	61	39	61
Mamouri et al. [65]	2004	Mashhad, Iran	Indo-Med (Iranian)	10	3	30	7	70	6	60	4	40	3	30	1	10	75	25
Pashapour and Golmaham- madlou [66]	2007	Urmia, Iran	Indo-Med (Iranian)	10	2	20	8	80										
Abdinejad et al. [34]	1996	Shriz, Iran	Indo-Med (Iranian)	30					15	50	15	50	2	7	13	43	13	87
Mirdad [63]	2002	Aseer, Saudi Arabia	Indo-Med (Saudi)	300	64	21.3	236	78.7	149	49.7	150	50.0	16	5.3	16	5.3	50	50
Kremli et al. [225]	2003	Riyadh, Saudi Arabia	Indo-Med (Saudi)	600	87	14.5	513	85.5	218	36.3	382	73.7	223	37.2	159	26.5	58.4	41.6
Mufti [226]	1988	Riyadh, Saudi Arabia	Indo-Med (Saudi) and others	79	34	44	44	56	36	46	41	54	29	37	12	15	53	37
Dğruel [60]	2008	Ankara, Turkey	Indo-Med (Turkish)	167	83	49.7	84	50.3										
Kutlu et al. [59]	1992	Konya, Turkey	Indo-Med (Turkish)	56	16	29	40	71	28	50	28	50	13	23	15	27	46	54
Kraus and Schwartzman [43]	1957	Fort Apache, Arizona	Nat. Am.	107	21	20	86	80	51	48	56	52						
Coleman [42]	1968	Fort Defiance AZ, Shiprock NM, Gallup NM	Nat. Am.	77	14	18	63	82	29	38	48	62	81	23	30	49	38	62
Walker [37]	1977	Island Lake, Manitoba	Nat. Am.	420	145	34.5	275	65.5	231	55.0	189	45.0	77	18.3	112	26.7	40.7	53.9
Rabin et al. [40]	1965	Many Farms District, Navajo Indian Reservation	Nat. Am.	31	6	19	25	81	6	19	25	81	15	48	10	32	60	40
MacKenzie et al. [227]	1960	Aberdeen and London, UK	Caucasian	134	20	15	114	85	31	23	103	77						
Wilkinson and Carter [228]	1960	London, England	Caucasian	149	17	11.4	132	88.6	42	28	107	28						
Noble et al. [114]	1978	Newcastle upon Tyne, England	Caucasian	271	60	22.1	211	77.9	103	38.0	168	62.0	39	14.4	129	47.6	23.2	76.8
Wray and Muddu [229]	1978	Stockport, England	Caucasian	130	48	37	82	63	39	30	91	70	56		113			
Heikkilä [105]	1984	Southern Finland	Caucasian	1035	208	20.1	827	79.9	342	33.0	693	67.0	225	21.7	559	54.0	28.7	71.3
Fredens- borg [95]	1976	Malmö, Sweden	Caucasian	548	118	21.5	430	78.5	314	57.3	234	42.7	143	21.6	91	16.6	61.1	38.9
Darmonov [131]	1996	Stara Zagora, Bulgaria	Caucasian	124	24	19.4	100	80.6	31	25.0	93	75.0	27	21.8	61	49.2	30.7	69.3
Tomáš [138]	1989	Bardejov, Slovakia	Caucasian	323	81	25.1	242	74.9										
Czéizel et al. [139]	1974	Békéscsaba, Hungary	Caucasian	523	77	14.7	446	85.3										
Poul et al. [135]	1992	Brno, Czechoslo- vakia	Caucasian	656	197	30.0	459	70.0	119	18.1	537	81.9	88	13.4	449	68.4	16.4	83.6
Vencálková and Janata [136]	2009	Liberec, Czech	Caucasian	452	63	14.3	390	45.7	113	25.0	339	75.0	131	29.0	208	46.0	38.6	61.4
Di Bella et al. [125]	1997	Sicily, Italy	Caucasian	51	8	16	43	84	3	6	48	94	9	18	39	76	19	81
Padilla-Esteban et al. [122]	1990	Madrid, Spain	Caucasian	1747	607	34.5	1140	65.3	648	37.1	1099	62.9	413	23.6	686	39.3	37.6	62.4
Romero et al. [230]	1989	Chile	Caucasian	97	13	13	84	86	66	68	30	31	15	16	15	16	50	50
Tijmes et al. [149]	1971	Llanquihue, Chile	Caucasian	137	33	24.1	104	75.9										
Robinson [231]	1968	New York State	Caucasian	339	68	20.1	271	79.9	70	21.9	249	78.1	81	25.4	168	52.7	32.5	67.5
Hazel and Beals [150]	1989	Portland, Oregon, USA	Caucasian	32	6	19	26	81	4	13	28	87	5	16	23	72	18	82
Hadlow [32]	1988	New Plymouth, New Zealand	Caucasian	172	16	9.3	162	94.2	87	50.6	85	49.4	11	6.4	74	43.0	12.9	87.1
Doig and Shannon [146]	1975	Christchurch, New Zealand	Caucasian	62	14	23	48	77	29	47	33	53	11	18	22	35	33	67
Paterson [67]	1976	South Australia	Caucasian and others	43	10	24	31	76	7	16	36	84	9	21	27	63	25	75
Yiv et al. [143]	1997	South Australia	Caucasian and others	206	48	23.3	158	76.7										
Bower et al. [44]	1987	Western Australia	Caucasian and others	437	101	23.1	336	76.9	165	37.8	223	51.0	65	14.9	158	36.2	29.1	70.9
Weighted Averages				**9717**	**2373**	**24.5**	**7317**	**75.5**	**2989**	**36.6**	**5169**	**63.4**	**1814**	**22.2**	**3229**	**39.6**	**36.0**	**64.0**

**Table 6:** Prevalence of adult acetabular dysplasia amongst different racial groups.

				Male			Female			Male and female			CEA*
Study	Year	Ethnicity	Location	No. Dysp.	No. Hips	%	No. Dysp.	No. Hips	%	No. Dysp.	No. Hips.	%	

Ali-Gombe et al. [382]	1996	African	Nigeria	3	126	2.4							<25°
Msamati et al. [383]	2003	African	Malawi	12	104	11.5	10	76	13.2	22	180	12.2	<25°
Skirving [384]	1981	African	—							4	162	2.5	<25°
*Average—Black*										**26**	**342**	**7.6**	
Yoshimura et al. [385]	1998	Caucasian	Britain	4	1303	0.3	4	195	2.1	8	1498	0.5	<25°
Croft et al. [386]	1991	Caucasian	Britain	26	2604	1.0							<20°
	1991	Caucasian	Britain	94	2604	3.6							<25°
Smith et al. [387]	1995	Caucasian	Britain				15	393	3.8	15	393	3.8	<25°
Lane et al. [388]	2000	Caucasian	USA				7	118	5.9	7	118	5.9	<30°
Jacobsen [389]	2007	Caucasian	Denmark	90	1352	6.7	152	2215	6.9	242	3567	6.8	<20°
Inoue et al. [390]	2000	Caucasian	France	10	549	1.8	13	234	5.6	23	783	2.9	<25°
Skirving [384]	1981	Caucasian	England							15	300	5.0	<25°
*Average—Caucasian*										**302**	**5161**	**5.9**	
Lau et al. [391]	1995	Indo-Malay (Chinese)	Hong Kong	89	999	8.9				89	999	8.9	<25°
	1995	Indo-Malay (Chinese)	Hong Kong	21	999	2.1				21	999	2.1	<20°
Hoaglund et al. [392]	1973	Indo-Malay (Chinese)	Hong Kong	1	248	0.4	3	252	1.2	4	500	0.8	<20°
	1973	Indo-Malay (Chinese)	Hong Kong	25	248	10.1	25	252	9.9	50	500	10.0	<25°
Yoshimura et al. [385]	1998	Indo-Malay (Japanese)	Japan	16	99	16.2	19	99	19.2	35	198	17.7	<25°
Inoue et al. [390]	2000	Indo-Malay (Japanese)	Japan	42	820	5.1	83	718	11.6	125	1538	8.1	<25°
Han et al. [393]	1998	Indo-Malay (Korean)	Korea	4	319	1.3	6	272	2.2	10	591	1.7	<20°
Moussa and Alomran [394]	2007	Indo-Med (Saudi)	Saudi Arabia							3	208	1.4	<25°
	2007	Indo-Med (Saudi)	Saudi Arabia							0	208	0.0	<20°
Goker et al. [395]	2004	Indo-Med (Turkish)	Turkey	13	130	10.0	4	54	7.4	17	184	9.2	<25°
	2004	Indo-Med (Turkish)	Turkey	3	130	2.3	0	54	0.0	3	184	1.6	<20°
Aktas et al. [396]	2000	Indo-Med (Turkish)	Turkey	5	202	2.5	3	293	1.0	8	495	1.6	≤22.5°
Umer et al. [397]	2006	Mixed—not stated	Singapore							19	522	3.6	<20°
*Average—Indo-Malay*										**327**	**4122**	**7.9**	<25°
	°									**57**	**3004**	**1.9**	<20°
Johnsen et al. [152]	2008	Sámi	Norway	21	150	14.0	35	165	21.2	56	315	**17.8**	<20°
	2008	Sámi	Norway	26	150	17.3	40	165	24.2	66	315	**21.0**	<25°

*CEA = center-edge angle of Wiberg [398].

**Table 7:** Archeological studies of hip dysplasia.

Study	Year	Archeological location	Era	M	F	U	B	No. DDH	No. Skeletons	Prevalence
*European peoples*										
Blondiaux and Millot [408]	1991	North and Eastern France	4th–13th century	3	3			6	700	8.6
Mafart et al. [409]	2007	Southern France	8th–17th century	0	9	5	4	9	900	10.0
Mitchell and Redfern [410]	2007	Spitalfields, London, UK	1100–1530	5	4	7	2	9	3290	2.7
Hawkes and Wells [411]	1983	Worthy Park, Hampshire, South England	5th–7th century					0	99	0
Eng et al. [412]	2009	Transylvania, Romania	1550–1700	0	2			2	70	28.6
Masnicová and Beňuš [413]	2003	Devín, Slovakia	9th–12th century	1		1		1	327	3.1
Maat et al. [414]	1995	Dordrecht, Netherlands	1375–1572					1	100	10.0
Lieverse [415]	2005	Cis-Baikal, Siberia	6800–1000 BC		1			1	271	3.7
Bourbou [416]	2003	Southern Greece	6th–7th century					0	225	0.0
Lieverse [417]	2008	Padova, Italy			1		1	1	213	4.7
Bisel [418]	1991	Herculaneum, Italy	79 AD		1		1	1	139	7.2
*Mid-Eastern/ Egyptian peoples*										
Goldstein et al. [419]	1976	Tel Sheva, Negev	200 BC					0	73	0.0
Ortner [420]	1979	Bâb edh-Dhrâ, Jordan	Early Bronze Age (3150–2200 BC)		1	1		1	92	10.9
Mathieson et al. [421]	1997	Gisr el-Mudir, Saqqara, Egypt	2890–2650 BC					1	24	41.7
*Indigenous peoples*										
Lahr and Bowan [422]	1992	Kechipawan, New Mexico	1300–1600					0	54	0.0
Pfeiffer [423]	1984	Uxbridge, Ontario	1490					1	312^∧^	3.2
Clabeaux [424]	1977	Fort Erie, Niagara River, Ontario	Pre- colonization					2	286	7.0
Wakefield et al. [425]	1937	Eastern Arkansas	Pre- colonization				1	1	100	10.0
Gregg et al. [426]	1981	Crow Creek, South Dakota	14th century	1	1		1	486	2.1	1
Miles [427]	1975	Mesa Verde, Colorado	750–1300 AD					0	179	0.0
Goldstein [428]	1957	Texas	800–1700 AD					0	146	0.0
Loveland et al. [429]	1985	Red River County, Texas	1100–1800 AD					1	75	
Wheeler [430]		El Morro Valley, New Mexico	13th Century					0	26	0.0
Merbs and Vestergaard [431]	1985	Sundown, Prescott, Arizona	1100–1200 AD					0	26	0.0
Drusini et al. [432]	1987	Maguana, Santo Domingo	late 15th century	3	2			5	108	46.3
Western Europe				8	16	12	6	25	5089	4.9
Eastern Europe				1	3	1	0	4	668	6.0
Mediterra- nean				0	2	0	2	2	577	3.5
Middle East				0	1	1	0	2	189	10.6
Indigenous				0	4	3	1	11	1798	6.1
All				9	26	17	9	44	8321	5.3

^∧^The skeletal remains derived from an ossuary make it impossible to reconstruct each individual from the commingled bones; the minimal number of individual skeletons is given. This results in a prevalence that cannot exceed the number calculated but may be less if there were more individuals represented in the ossuary.
